# Short- and Long-term Effects of a Mobile Phone App in Conjunction With Brief In-Person Counseling on Physical Activity Among Physically Inactive Women: The mPED Randomized Clinical Trial

**DOI:** 10.1001/jamanetworkopen.2019.4281

**Published:** 2019-05-24

**Authors:** Yoshimi Fukuoka, William Haskell, Feng Lin, Eric Vittinghoff

**Affiliations:** 1 Department of Physiological Nursing, Institute for Health & Aging, School of Nursing, University of California, San Francisco; 2 Stanford Prevention Research Center, Stanford University, Palo Alto, California; 3 Department of Epidemiology & Biostatistics, University of California, San Francisco

## Abstract

**Importance:**

Mobile phone applications (apps) and activity trackers allow researchers to remotely deliver an intervention and monitor physical activity but have not been rigorously evaluated for longer periods.

**Objective:**

To determine whether a mobile phone–based physical activity education app, in conjunction with brief in-person counseling, increases and then maintains levels of physical activity.

**Design, Setting, and Participants:**

In this parallel randomized clinical trial, community-dwelling physically inactive women recruited between May 2011 and April 2014 were randomized in equal proportions into the control (n = 69), regular (n = 71), and plus (n = 70) groups. Data were analyzed using intention to treat from September 16, 2016, through June 30, 2018.

**Interventions:**

The regular and plus groups were instructed to use the app on their mobile phone and an accelerometer every day for 3 months and attend brief in-person counseling. During the 6-month maintenance period, the plus group continued to use the app and accelerometer, while the regular group stopped using the app but continued using the accelerometer. The control group used the accelerometer throughout.

**Main Outcomes and Measures:**

The primary and secondary outcomes were daily accelerometer-measured total steps and time spent in moderate to vigorous physical activity (MVPA).

**Results:**

The 210 participants had a mean (SD) age of 52.4 (11.0) years. At baseline, the mean (SD) daily total steps by accelerometer in the control, regular, and plus groups were 5384 (2920), 5063 (2526), and 5837 (3235), respectively. During the 3-month intervention period, daily steps and MVPA increased in the combined regular and plus groups compared with the control group (between-group differences, 2060 steps per day; 95% CI, 1296-2825 steps per day; *P* < .001 and 18.2 min/d MVPA; 95% CI, 10.9-25.4 min/d MVPA; *P* < .001). During the subsequent 6-month maintenance period, mean activity level remained higher in the combined plus and regular groups than among controls (between-group difference, 1360 steps per day; 95% CI, 694-2026 steps per day; *P* <. 001), but trends in total daily steps and MVPA were similar in the plus and regular groups.

**Conclusions and Relevance:**

In this trial, the intervention groups substantially increased their physical activity. However, use of both the app and accelerometer for an additional 6 months after the initial 3-month intervention did not help to maintain increases in physical activity compared with continued use of the accelerometer alone.

**Trial Registration:**

ClinicalTrials.gov identifier: NCT01280812

## Key Points

**Question:**

Does use of a mobile phone–based physical activity education application (app) in conjunction with brief in-person counseling result in an increase of accelerometer-measured physical activity for 3 months and maintaining activity for an additional 6 months?

**Findings:**

In this randomized clinical trial of 210 community-dwelling physically inactive women, the intervention achieved a statistically and clinically significant increase in total steps and time spent performing moderate to vigorous physical activity compared with the control group in the first 3 months. However, the group who continued use of the app, as compared with the group who discontinued app use, experienced no statistically significant effect on maintaining the increased activity in the following 6 months.

**Meaning:**

The combination of a mobile phone app and brief in-person counseling increased objectively measured physical activity over 3 months, but use of the app for an additional 6 months did not help to maintain increased activity.

## Teaser

This randomized clinical trial evaluates the effectiveness of a mobile phone app combined with brief in-person counseling for increasing physical activity levels among inactive women.

## Introduction

Despite the many health benefits of physical activity, most American adults do not meet the current physical activity recommendations when objective measures are used to assess physical activity.^1,2,3^ In addition, women are less likely to be physically active than men across all age groups.^1,4^ Mobile phone applications (apps) and physical activity trackers designed to increase and maintain physical activity have rapidly gained popularity and may be cost-effective for promotion of physical activity.

However, a number of factors have limited the assessment of digital technology–based physical activity interventions.^5^ First, studies are frequently short, and only a few have examined maintenance of physical activity after the initial intervention. Second, sample sizes have been small, and only self-reported measures of physical activity have been used in some studies.^6,7^ Third, although app use is associated with behavior change, app use statistics or user engagement information are seldom reported.^6^ To address these limitations, we designed a 9-month, 3-group, randomized clinical trial to assess a mobile phone–based physical activity education (mPED) program. Physical activity was objectively measured using an accelerometer in all 3 groups. For the first 3 months, the regular and plus groups received an identical intervention, including the mPED app and brief in-person counseling. In the following 6 months, the plus group continued to use both the app and accelerometer, while the regular group used the accelerometer only.

## Methods 

### Study Design and Participants

The mPED study was an unblinded, parallel randomized clinical trial conducted with 3 groups (control, regular, and plus). The trial consisted of a 3-week run-in period, a 3-month intervention period using the app and counseling to increase physical activity, and a 6-month maintenance period using the app to maintain activity. Detailed methods are published elsewhere.^3,8,9,10^ This study followed the Consolidated Standards of Reporting Trials (CONSORT) reporting guideline. The trial protocol is available in Supplement 1 and was approved by the institutional review board at the University of California, San Francisco, and by the safety monitoring board appointed by the research team. Written informed consent was obtained from all participants. Participants were recruited between May 2011 and April 2014 using media advertising, mass mailing to residents who met age and sex inclusion criteria, and flyer posting at medical clinics, churches, universities, and community centers in the San Francisco Bay Area. In brief, eligibility criteria for inclusion in the study were female sex, age from 25 to 65 years, body mass index (BMI; calculated as weight in kilograms divided by height in meters squared) of 18.5 to 43.0, physically inactive at work and/or during leisure time based on the Stanford Brief Activity Survey,^11^ intent to be physically active, access to a home telephone or mobile phone, ability to speak and read English, no medical conditions or physical problems that required special attention in an exercise program, no current participation in other lifestyle modification programs, and no mild cognitive impairment as determined by the Mini-Cog test.^12^

### Run-in Period and Randomization

The run-in period was used to determine the baseline average daily steps, other accelerometer-measured physical activity, and whether the participant was able to comply with requirements of the study, as defined by at least 80% response rates to daily messaging, 80% use of the daily activity diary, and wearing the accelerometer for at least 8 hours per day. During the run-in period, the Omron Active Style Pro HJA-350IT accelerometer was set to record and store physical activity but did not display physical activity information (eg, steps). At the end of the run-in period, participants meeting the compliance requirements were randomly assigned in equal proportions to the control, regular, and plus groups using randomly permuted blocks of randomly selected block sizes of 3, 6, and 9.

### Control vs Interventions

Control group members were asked to use an accelerometer for the entire study period but did not receive any physical activity intervention. In the 3-month intervention period, the regular and plus groups received the identical physical activity intervention, consisting of brief in-person counseling sessions at randomization, 6 weeks, and 3 months and the mPED app. A detailed description of both components was published previously.^8^ The in-person counseling sessions included 7 domains: (1) overview of the physical activity program and tailored short- and long-term goal setting based on each participant’s baseline physical activity data, (2) education about duration and intensity of brisk walking and the health benefits of physical activity, (3) identification of barriers to increasing physical activity and development of strategies to overcome these barriers, (4) value and identification of social support while increasing physical activity, (5) relapse prevention, (6) education about healthy diet and weight maintenance, and (7) physical activity safety. A written individualized physical activity plan was developed during the initial in-person counseling session immediately after randomization and then reevaluated at the 6-week and 3-month visits. The mPED app developed by the research team has 2 main functions: (1) a daily message or video clip and (2) a daily diary. The daily messages and video clips reinforced the 7 domains addressed in the brief in-person intervention. A preprogrammed daily message or video clip was sent once per day at a predetermined time between 11 am and 3 pm. The daily physical activity diary was accessible between 7 pm and midnight. If no diary entry was made by 8:30 pm, an automated text message was sent to remind the participant to record her total daily steps and the type and duration of physical activities performed. An automated text message was also sent if a participant did not use the app for 3 consecutive days. The app also included “summary,” “help,” “talk to us,” and “weekly goals” menu options. The summary option included the material provided in the brief in-person counseling session; the help option listed the research office contact information; and the talk to us option allowed the participant to directly send a text message to researchers from the application. Activity goals, displayed in the weekly goals option, were automatically increased by 20% each week, relative to the participant’s run-in average, until a goal of 10 000 steps per day, 7 days per week, was reached. At 3 months, the mPED app was removed from the mobile phones of the regular group, while the plus group kept the mPED app and was encouraged to continue using the physical activity diary; both groups continued to use the accelerometer to measure activity. The rationale for testing 2 different maintenance interventions (regular vs plus) was to understand maintenance strategies in terms of a dose response to self-monitoring and feedback, which are important components of physical activity maintenance.

### Fidelity of the Intervention

The principal investigator trained nonmedical research staff and observed at least the first 2 in-person interventions. All in-person sessions were digitally recorded and downloaded to a password-protected research computer to check fidelity of the intervention. The length of the in-person session and the number and type of questions participants asked were recorded by research staff; these data were periodically analyzed by the data specialist throughout the trial period.

### Measurements

All baseline measures were collected at a screening/baseline visit or during the run-in period prior to randomization. The primary and secondary outcomes were total daily steps and duration of moderate to vigorous physical activity (MVPA), measured every day for 9 months using the Omron Active Style Pro HJA-350IT triaxial accelerometer. This accelerometer has been validated before, and a detailed description was published previously.^13,14^ This accelerometer was programed to collect daily steps and physical activity intensity (metabolic equivalent values [METs]). The mean intensity value of a 1-minute epoch was calculated as the average value of six 10-second epochs. The METs determined by this accelerometer are closely correlated with METs calculated using energy expenditure measured by indirect calorimetry. Using the Compendium of Physical Activities,^15,16^ moderate physical activity was defined as greater than or equal to 3 but fewer than 6 METs and vigorous activity was defined as 6 METs or greater. The accelerometer automatically reset the count each day at midnight and allowed participants to view their counts for the past 7 days. Participants were instructed to place the accelerometer on the waist in line with the middle of the thigh of their dominant leg and wear it from the time they got up in the morning until they went to bed at night every day except when showering, bathing, swimming, or sleeping at night. Activity data from the most recent 150 days were automatically stored and directly downloaded to a computer in our research office. The criterion for acceptable accelerometer data was that the downloaded data must show that the participant wore it at least 8 hours per day 4 or more days per week. In addition, self-reported physical activity was assessed at baseline, 3 months, and 9 months using the interviewer-administered 7-Day Physical Activity Recall questionnaire.^17^ Other study measures assessed during study visits included the modified Self-Efficacy for Physical Activity Survey,^18^ Social Support and Exercise Survey,^19^ 12-Item Short-Form Health Survey,^20^ Barriers to Being Active Quiz,^21^ and the Center for Epidemiological Studies Depression Scale.^22^ Sociodemographic characteristics, including self-identified race/ethnicity, were collected at baseline.

### Statistical Analysis

The planned overall sample size of 192 participants, randomized in equal proportions to the control, regular, and plus groups, was determined to provide 90% power in 2-sided tests with a type I error rate of 5% to detect a difference between the regular and plus groups of 1100 steps per day in the average change from 3 to 9 months, after accounting for 12% loss to follow-up by 3 months and an additional loss of 13% during the 6-month maintenance period. In comparisons of the regular and plus with the control group of changes in steps during the first 3 months, the overall sample of 192 was estimated to provide 90% power to detect between-group differences of approximately 1000 steps per day, and greater than 99% power to detect the hypothesized difference of 2200 steps per day. Sample size calculations accounted for the intraclass correlations of the repeated outcomes as well as loss to follow-up.

Baseline characteristics of the 3 groups were compared using parametric and nonparametric tests, as appropriate. We used linear mixed models (LMMs) to assess effects of the interventions on trends in daily measurements of step counts, steps per hour, moderate physical activity, vigorous physical activity, and MVPA. In the LMMs for intervention effects in the first 3 months, we used a linear spline in days since randomization to allow for an immediate increase in each group followed by accumulating differences over the remainder of this period. Treatment effects were captured by the fitted between-group difference at 3 months, net of any baseline difference, calculated using a linear combination of the LMM coefficients. To account for within-participant correlation of the repeated outcomes, random intercepts and linear spline components were included in the LMM. Moderation of the primary intervention effects by age greater than or equal to 55 years and BMI greater than or equal to 30 was assessed by adding interactions to the LMM as appropriate. We also used LMMs to assess the effects of the intervention during the 6-month maintenance period from 3 to 9 months, in this case assuming simpler linear trends in the regular and plus groups; a first LMM was used to assess the mean difference between groups over the 6 months, and a second to assess the between-group difference in mean change per month. Two-sided *P* values less than .05 were considered statistically significant. Analyses were implemented using SAS statistical software version 9.4 (SAS Institute) and Stata statistical software version 15.1 (StataCorp LLC). Data collected between September 16, 2016, and June 30, 2018, were analyzed.

## Results

### Participants and Baseline Characteristics

**Figure 1.**
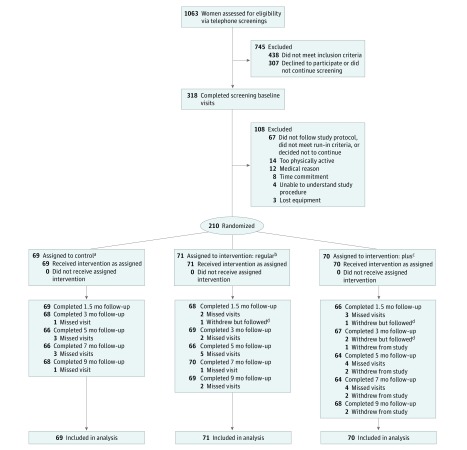
Study Flow Diagram ^a^The control group used the accelerometer only for 9 months with no intervention. ^b^The regular group completed the 3-month physical activity intervention and 6-month accelerometer maintenance intervention. ^c^The plus group completed the 3-month physical activity intervention and 6-month mobile phone diary maintenance intervention with accelerometer. ^d^Participants were counted as “withdrew but followed” on the day they withdrew. Each follow-up visit after the date of withdrawal, they are included as “completed.”

We initially screened 1063 individuals via telephone, of whom 318 completed a screening/baseline visit (Figure 1). After the run-in period, a total of 210 physically inactive community-dwelling women were randomized as follows: 69 to the control group, 71 to the regular group, and 70 to the plus group. All participants in the regular and plus groups received initial brief in-person counseling and the mPED app immediately after randomization. The 9-month retention rates were 98.6% (68 of 69) for the control group, 97.1% (69 of 71) for the regular group, and 97.1% (68 of 70) for the plus group. The overall retention rate was 97.6%. Table 1 shows baseline characteristics and physical activity of the participants. The mean (SD) age was 52.4 (11.0) years, 56.7% (119 of 210) self-identified as non-Hispanic white, 75.3% (158 of 210) had at least a college education, 74.3% (158 of 210) had a paid job, and the mean (SD) BMI was 29.9 (6.2). No significant differences in baseline characteristics were observed among the 3 groups (Table 1).

**Table 1.** Baseline Information by Treatment Group for 210 Participants

Characteristic	No. (%)		
	Control (n = 69)	Regular (n = 71)	Plus (n = 70)
Sociodemographic factors			
Age, mean (SD), y	51.7 (10.1)	53.5 (11.7)	52.0 (11.2)
Age group			
<40 y	9 (13.0)	11 (15.5)	10 (14.3)
40-49 y	19 (27.5)	9 (12.7)	15 (21.4)
50-59 y	23 (33.3)	25 (35.2)	26 (37.1)
≥60 y	18 (26.1)	26 (36.6)	19 (27.1)
Race/ethnicity			
African American	9 (13.0)	4 (5.6)	4 (5.7)
Hispanic/Latino	3 (4.3)	4 (5.6)	6 (8.6)
Asian	13 (18.8)	14 (19.7)	14 (20.0)
White (non-Hispanic)	35 (50.7)	42 (59.2)	42 (60.0)
>1 race	9 (13.0)	7 (9.9)	4 (5.7)
Education			
Completed high school or some college	24 (34.8)	13 (18.3)	15 (21.4)
Completed 4-y college	23 (33.3)	33 (46.5)	30 (42.9)
Completed graduate school	22 (31.9)	25 (35.2)	25 (35.7)
Annual household income before tax, $			
≤20 000	5 (7.2)	3 (4.2)	4 (5.7)
20 001-40 000	8 (11.6)	4 (5.6)	8 (11.4)
40 001-75 000	13 (18.8)	19 (26.8)	18 (25.7)
>75 000	36 (52.2)	38 (53.5)	37 (52.9)
Decline to state	7 (10.1)	5 (7.0)	2 (2.9)
Unknown	0	2 (2.8)	1 (1.4)
Marital status			
Never married	21 (30.4)	18 (25.4)	25 (35.7)
Currently married or cohabitating	40 (58.0)	36 (50.7)	31 (44.3)
Divorced or widowed	8 (11.6)	17 (23.9)	14 (20.0)
Employed for pay full or part time	47 (68.1)	58 (81.7)	51 (72.9)
Previous pedometer use	34 (49.3)	35 (49.3)	40 (57.1)
Drives a car ≥1 time/wk	60 (87.0)	61 (85.9)	55 (78.6)
Has a dog	18 (40.9)	13 (18.3)	13 (18.6)
Participated in a diet or weight loss plan	44 (63.8)	45 (63.4)	43 (61.4)
Has a gym membership	18 (26.1)	20 (28.2)	21 (30.0)
Self-reported cardiovascular risk factors			
Body mass index^a^	30.2 (5.8)	29.6 (6.3)	29.8 (6.3)
Body mass index category^a^			
<25	16 (23.2)	21 (29.6)	18.0 (25.7)
25-30	18 (26.1)	15.0 (21.1)	22 (31.43)
>30	35 (50.7)	35 (49.3)	30 (42.9)
Current smoker	2 (2.9)	0	2 (2.9)
Reached menopause	39 (56.5)	45 (63.4)	40 (57.1)
High blood pressure	17 (24.6)	15 (21.1)	23 (32.9)
High total cholesterol	22 (31.9)	24 (33.8)	25 (35.7)
High glucose or diabetes	4 (5.8)	4 (5.6)	8 (11.4)
Center for Epidemiologic Studies Depression Scale score >16 or taking antidepressant	22 (31.9)	24 (33.8)	26 (37.1)
Computer use, h/wk	26.4 (20.6)	27.9 (18.7)	28.2 (16.2)
Television use, h/wk	13.6 (11.3)	14.7 (9.4)	13.6 (10.5)

^a^ Calculated as weight in kilograms divided by height in meters squared.

### Intervention Adherence

Regular and plus group participants received brief in-person counseling sessions at the randomization visit (141 of 141 [100%]), 6-week visit (133 of 141 [94.3%]), and 3-month visit (136 of 141 [96.5%]) in addition to the mPED app. The mean (SD) duration of the initial in-person counseling time and the mean (SD) number of questions from the participants did not differ between the regular and plus groups (counseling time, 29.0 [7.1] minutes vs 28.6 [6.1] minutes and number of questions, 2.6 [3.6] vs 2.2 [2.0]). Furthermore, the mean (SD) adherence to daily messages or video clips and daily physical activity diary via the study app during the initial 3 months did not differ between the regular and plus groups (85.5% [15.2%] vs 85.7% [15.4%]). From 3 to 9 months, only participants in the plus group were asked to use the daily physical activity diary via the mPED app and the mean (SD) adherence was 68.4% (28.2%). Adherence rates were penalized for malfunctions of the mPED app and/or study server.

### Intervention Effects

**Figure 2.**
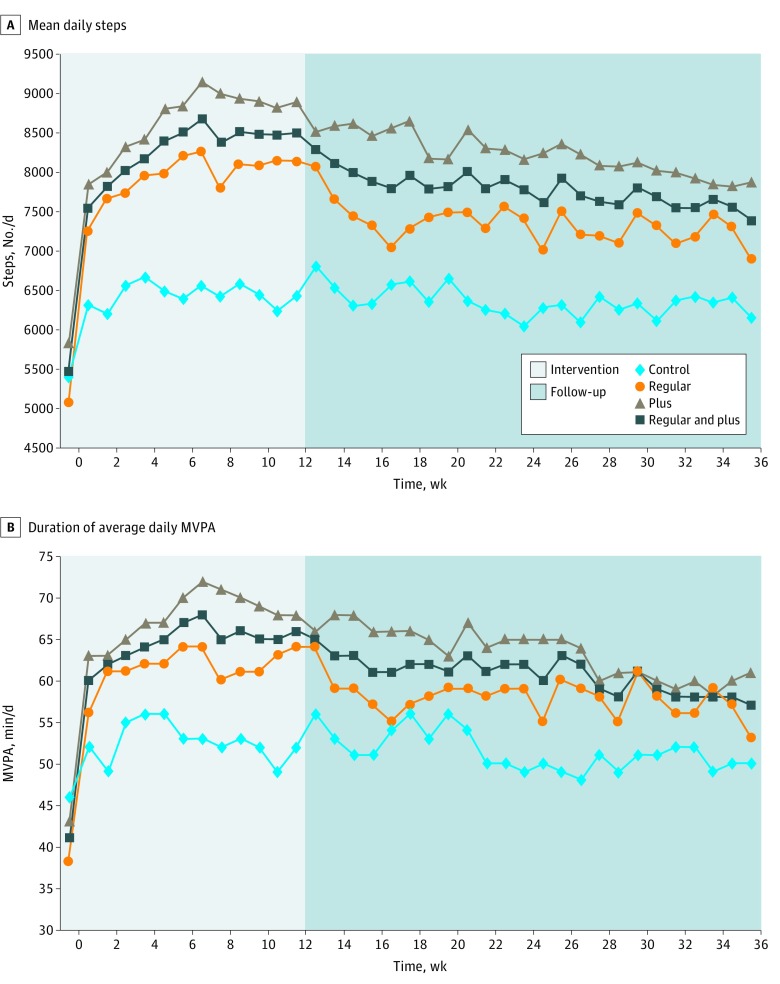
Changes in Mean Daily Steps and Mean Daily Moderate to Vigorous Physical Activity (MVPA) by Treatment Group and Week The weekly daily average is shown for participants in the control group, the regular intervention group, and the plus intervention group, as well as combined results for the 2 intervention groups.

At baseline, mean (SD) daily total steps by accelerometer in the control, regular, and plus groups were 5384 (2920), 5063 (2526), and 5837 (3235), respectively (Table 2). Although there was no difference in the average baseline hourly steps between control and combined regular and plus groups, the number of hourly steps in the plus group was slightly greater than the regular group. Figure 2 shows the trends of total daily steps and MVPA over the 9-month study period. Compared with controls, regular and plus groups had an increase in daily total steps (mean difference, 2060 steps per day; 95% CI, 1296-2825 steps per day; *P* < .001) and MVPA (mean difference, 18.2 min/d; 95% CI, 10.9-25.4 min/d; *P* < .001) (Table 3). During the subsequent 6-month maintenance period, average activity level remained higher in the combined plus and regular groups than among controls (between-group difference, 1360 steps per day; 95% CI, 694-2026 steps per day; *P* < .001). During the 6-month maintenance period, trends in daily steps and MVPA in the regular and plus groups were similar. Results for total daily steps were similar in a per-protocol analysis restricted to observations with valid accelerometer data. Subgroup analyses gave no evidence that age (age ≥55 or <55 years) and baseline BMI (≥30 or <30) moderated the effects of the intervention (eTable 1 in Supplement 2). Secondary outcomes, including the Barriers to Being Active Quiz, Center for Epidemiological Studies Depression Scale, modified Self-efficacy for Physical Activity Survey, Social Support and Exercise Survey, and 12-Item Short-Form Health Survey physical component scores, significantly improved from baseline to 3 months in the combined regular and plus groups compared with the control group (eTable 2 in Supplement 2) but did not differ between the regular and plus groups at 9 months. There was no evidence for intervention effects on the 12-Item Short-Form Health Survey mental component scores. There were no missing values for baseline measurements. Physical activity outcomes measured using the accelerometer, including daily step counts and MVPA, were missing for approximately 12% of follow-up days during the 9-month trial period. Self-reported physical activity outcome was missing at less than 1% of completed visits.

**Table 2.** Baseline Accelerometer-Measured and Self-Reported Physical Activity for 210 Participants

Physical Activity	Mean (SD)			*P* Value	
	Control (n = 69)	Intervention		(Intervention vs Control)	(Regular vs Plus)
		Regular (n = 70)	Plus (n = 71)		
Accelerometer					
Steps/d	5384 (2920)	5063 (2526)	5837 (3235)	.40	.08
Steps/h	403 (214)	375 (180)	431 (226)	.46	.04
Moderate physical activity, min/d	45.6 (33.3)	37.6 (23.9)	42.8 (28.5)	.56	.30
Vigorous physical activity, min/d	0.14 (0.82)	0.44 (2.4)	0.70 (3.31)	.03	.70
Moderate to vigorous physical activity, min/d	45.7 (33.4)	38.0 (24.4)	43.5 (29.6)	.62	.30
Self-reported					
7-d physical activity recall, kcal/kg/d^a^	32.9 (1.4)	32.9 (1.1)	33.1 (1.1)	.43	.39

^a^ The interviewer-administered 7-Day Physical Activity Recall questionnaire was used to obtain self-reported activity.

**Table 3.** Intervention Effects on Accelerometer-Measured and Self-Reported Physical Activity Outcomes

Physical Activity Outcome	Baseline to Month 3		Month 3 to Month 9					
	Net Effect, Intervention vs Control (95% CI)	*P* Value	Difference in Trends, Plus vs Regular (95% CI)^a^	*P* Value	Difference in Level, Plus vs Regular (95% CI)^a^	*P* Value	Difference in Level, Intervention vs Control (95% CI)^a^	*P* Value
**Intention-to-Treat**								
Accelerometer								
Steps/d, No.	2060 (1296 to 2825)	<.001	−245 (−907 to 417)	.47	900 (94 to 1707)	.03	1360 (694 to 2026)	<.001
Steps/h, No.	139 (86.4 to 191.0)	<.001	−20.8 (−68.1 to 26.4)	.39	62.2 (8.2 to 116.1)	.02	83.7 (38.7 to 128.8)	<.001
Moderate physical activity, min/d	16.3 (9.4 to 23.2)	<.001	−4.9 (−11.2 to 1.4)	.13	4.6 (−2.6 to 11.7)	.21	7.3 (1.2 to 13.5)	.02
Vigorous physical activity, min/d	1.8 (0.7 to 3.0)	.002	−0.3 (−1.4 to 0.9)	.68	0.3 (−0.7 to 1.4)	.55	1.1 (0.4 to 1.9)	.004
Moderate to vigorous physical activity, min/d	18.2 (10.9 to 25.4)	<.001	−5.2 (−11.9 to 1.4)	.13	4.9 (−2.7 to 12.5)	.21	8.4 (2.0 to 14.9)	.01
Self-report								
7-d physical activity recall, kcal/kg/d^b^	0.84 (0.34 to 1.33)	.001	0.64 (−0.04 to 1.32)	.07	0.06 (−0.52 to 0.64)	.84	0.45 (−0.06 to 0.96)	.08
**Per-Protocol**								
Accelerometer								
Steps/d, No.	2077 (1310 to 2843)	<.001	−224 (−888 to 440)	.51	903.0 (97.1 to 1709.3)	.03	1366 (700 to 2032)	<.001
Steps/h, No.	139 (87 to 192)	<.001	−19.6 (−66.9 to 27.7)	.42	62.3 (8.5 to 116.2)	.02	84.0 (39.0 to 129.0)	<.001

^a^ Differences on level and trend reflect comparisons of the mean level of the outcome and of the fitted change per month from month 3 to month 9, respectively.

^b^ The interviewer-administered 7-Day Physical Activity Recall questionnaire was used to obtain self-reported activity.

### Adverse Events

No differences in hospital admissions, emergency department or urgent care facility visits, or other adverse events were observed between the control, and regular, and plus groups during the 3-month intervention period and among the 3 groups during the 6-month maintenance period (eTable 3 in Supplement 2).

## Discussion

In this trial, a 3-month intervention using an app and brief in-person counseling resulted in a net increase of approximately 2000 steps per day (equivalent to approximately 1 mile or 20 minutes walking per day) and 18 minutes of MVPA per day. These increases helped participants toward meeting the current physical activity recommendation.^23^ During the maintenance period from 3 to 9 months, mean daily steps and MVPA remained higher in the intervention groups than among controls, but declined in the regular and plus groups at similar rates, thus demonstrating that continued use of the mPED app in the plus group did not improve maintenance of the initial increase in physical activity.

To our knowledge, this trial is one of the first studies to examine the effect of an app-based intervention on increasing and maintaining objectively measured daily physical activity, with excellent retention of participants and collection of accelerometer data. According to recent systematic reviews, up to one-half of all app-based physical activity interventions for adults were ineffective^6,7^; among trials using an objective measurement of physical activity, the proportion of ineffective interventions is even greater. These negative findings may, in part, reflect relatively brief interventions, with an average duration of 10 weeks.^6^

There are several potential explanations for the initial effectiveness of the mPED intervention. First, it is known that tailoring an intervention to a targeted population is critical for behavioral changes.^24^ In this trial, the mPED app included daily messages and video clips and in-person counseling sessions specifically designed for physically inactive women based on our literature review and pilot studies.^25,26^ In particular, it is important for women to be able to engage in the intervention at home, as they tend to have other competing responsibilities. Second, behavioral change strategies known to be effective, including individualized short-term goal setting and feedback, self-monitoring, social support, relapse prevention, and automated reminders, were incorporated into the app and in-person counseling sessions. According to a systematic review of studies using pedometers to increase physical activity, having a daily step goal, using a step diary (self-monitoring), and having an intervention other than in the workplace were significant key predictors of increased physical activity.^27^ There are many commercially available physical activity apps, but few apps have systematically incorporated evidence-based behavior change strategies.^28,29^

In contrast, we found no evidence that continued use of the mPED app in the plus group helped to maintain the increases in physical activity achieved in the combined plus and regular groups during the first 3 months of maintenance. One possibility is that individuals are able to master skills during the initial intervention period so that the app is no longer needed, and the physical activity tracker suffices to maintain activity. In a recent large trial^30,31^ in Spain, accelerometer-measured total daily steps in both the app with in-person counseling intervention group and the counseling-alone group decreased from baseline to 3 months as well as during the 12-month follow-up period. The mean changes between the 2 groups did not differ. The findings of this large trial are inconsistent with our findings, and this could be due to a very large number of total steps at baseline in the Spanish trial.

The findings of our prespecified subgroup analyses suggested that age and BMI did not modify either the initial or maintenance effects of the intervention, suggesting that our findings may be broadly applicable. Generally, older adults are less likely to own a smartphone^32^ and have lower levels of technology literacy than younger adults, suggesting that digital technology–based interventions are more suitable for younger adults. However, the evidence appears to support the concept that older adults had an equal or greater level of engagement in app use compared with younger adults.^30,33,34^ Given the rapid adoption of smartphones among older adults,^35^ the findings of the subgroup analyses also have important implications for developing age-targeted interventions in the future.

### Limitations

This trial has several limitations. First, the mPED sample included only female adults who were motivated to become physically active, which may limit generalizability of the findings. However, the mean baseline number of daily steps in this trial was very similar to the US National Health and Nutrition Examination Survey female adult data.^36^ Second, the triaxial accelerometer used in this trial was not able to record water activities (eg, swimming) or might not be able to completely capture some activities (eg, household chores using upper body movements, yoga), which might result in underestimation of activity. However, like other studies, the proportion of participants who engaged in these activities in this trial was relatively small,^3^ and brisk walking, which the accelerometer does measure, was the target of the intervention. In addition, because the intervention consisted of 2 components (brief in-person sessions and daily app use), the independent effect of daily app use cannot be determined.

## Conclusions

In this trial of 210 community-dwelling physically inactive women, our 3-month app-based and counseling intervention achieved a significant initial increase in physical activity among the participants. However, use of the app for an additional 6 months did not help to maintain physical activity compared with using the accelerometer alone. Methods for maintaining gains achieved by app-based physical activity interventions require further development.

## Notes

Supplement 1. **Trial Protocol**

Supplement 2. **eTable 1.** Subgroup Analyses of Age and Body Mass Index (BMI) in Daily Total Steps and Moderate to Vigorous Physical Activity (MVPA) **eTable 2.** Other Self-Reported Measures **eTable 3.** Safety Data Summary

Supplement 3. **Data Sharing Statement**

## References

[1] Troiano RP, Berrigan D, Dodd KW, Mâsse LC, Tilert T, McDowell M Physical activity in the United States measured by accelerometer. Med Sci Sports Exerc. 2008;40(1):-. doi:10.1249/mss.0b013e31815a51b3 18091006

[2] Shiroma EJ, Cook NR, Manson JE, Buring JE, Rimm EB, Lee IM Comparison of self-reported and accelerometer-assessed physical activity in older women. PLoS One. 2015;10(12):e0145950. doi:10.1371/journal.pone.0145950 26713857 PMC4694656

[3] Fukuoka Y, Haskell W, Vittinghoff E New insights into discrepancies between self-reported and accelerometer-measured moderate to vigorous physical activity among women—the mPED trial. BMC Public Health. 2016;16(1):761. doi:10.1186/s12889-016-3348-7 27514368 PMC4982411

[4] Kao MC, Jarosz R, Goldin M, Patel A, Smuck M Determinants of physical activity in America: a first characterization of physical activity profile using the National Health and Nutrition Examination Survey (NHANES). PM R. 2014;6(10):882-892. doi:10.1016/j.pmrj.2014.03.004 24631950

[5] Grady A, Yoong S, Sutherland R, Lee H, Nathan N, Wolfenden L Improving the public health impact of eHealth and mHealth interventions. Aust N Z J Public Health. 2018;42(2):118-119. doi:10.1111/1753-6405.12771 29384248

[6] Schoeppe S, Alley S, Van Lippevelde W, Efficacy of interventions that use apps to improve diet, physical activity and sedentary behaviour: a systematic review. Int J Behav Nutr Phys Act. 2016;13(1):127. doi:10.1186/s12966-016-0454-y 27927218 PMC5142356

[7] Stuckey MI, Carter SW, Knight E The role of smartphones in encouraging physical activity in adults. Int J Gen Med. 2017;10:293-303. doi:10.2147/IJGM.S134095 28979157 PMC5602432

[8] Fukuoka Y, Komatsu J, Suarez L, The mPED randomized controlled clinical trial: applying mobile persuasive technologies to increase physical activity in sedentary women protocol. BMC Public Health. 2011;11:933. doi:10.1186/1471-2458-11-933 22168267 PMC3295748

[9] Fukuoka Y, Zhou M, Vittinghoff E, Haskell W, Goldberg K, Aswani A Objectively measured baseline physical activity patterns in women in the mPED trial: cluster analysis. JMIR Public Health Surveill. 2018;4(1):e10. doi:10.2196/publichealth.9138 29391341 PMC5814604

[10] Fukuoka Y, Lindgren TG, Mintz YD, Hooper J, Aswani A Applying natural language processing to understand motivational profiles for maintaining physical activity after a mobile app and accelerometer-based intervention: the mPED randomized controlled trial. JMIR Mhealth Uhealth. 2018;6(6):e10042. doi:10.2196/10042 29925491 PMC6031900

[11] Taylor-Piliae RE, Norton LC, Haskell WL, Validation of a new brief physical activity survey among men and women aged 60-69 years. Am J Epidemiol. 2006;164(6):598-606. doi:10.1093/aje/kwj248 16840522

[12] Borson S, Scanlan J, Brush M, Vitaliano P, Dokmak A The mini-cog: a cognitive ‘vital signs’ measure for dementia screening in multi-lingual elderly. Int J Geriatr Psychiatry. 2000;15(11):1021-1027. doi:10.1002/1099-1166(200011)15:11<1021::AID-GPS234>3.0.CO;2-6 11113982

[13] Oshima Y, Kawaguchi K, Tanaka S, Classifying household and locomotive activities using a triaxial accelerometer. Gait Posture. 2010;31(3):370-374. doi:10.1016/j.gaitpost.2010.01.005 20138524

[14] Ohkawara K, Oshima Y, Hikihara Y, Ishikawa-Takata K, Tabata I, Tanaka S Real-time estimation of daily physical activity intensity by a triaxial accelerometer and a gravity-removal classification algorithm. Br J Nutr. 2011;105(11):1681-1691. doi:10.1017/S0007114510005441 21262061

[15] Ainsworth BE, Haskell WL, Leon AS, Compendium of Physical Activities: classification of energy costs of human physical activities. Med Sci Sports Exerc. 1993;25(1):71-80. doi:10.1249/00005768-199301000-00011 8292105

[16] Ainsworth BE, Haskell WL, Whitt MC, Compendium of Physical Activities: an update of activity codes and MET intensities. Med Sci Sports Exerc. 2000;32(9)(suppl):S498-S504. doi:10.1097/00005768-200009001-00009 10993420

[17] Sallis JF, Haskell WL, Wood PD, Physical activity assessment methodology in the Five-City Project. Am J Epidemiol. 1985;121(1):91-106. doi:10.1093/oxfordjournals.aje.a113987 3964995

[18] Marcus BH, Selby VC, Niaura RS, Rossi JS Self-efficacy and the stages of exercise behavior change. Res Q Exerc Sport. 1992;63(1):60-66. doi:10.1080/02701367.1992.10607557 1574662

[19] Sallis JF, Grossman RM, Pinski RB, Patterson TL, Nader PR The development of scales to measure social support for diet and exercise behaviors. Prev Med. 1987;16(6):825-836. doi:10.1016/0091-7435(87)90022-3 3432232

[20] Ware J Jr, Kosinski M, Keller SDA A 12-item short-form health survey: construction of scales and preliminary tests of reliability and validity. Med Care. 1996;34(3):220-233. doi:10.1097/00005650-199603000-00003 8628042

[21] Centers for Disease Control and Prevention Barriers to Being Active Quiz. http://www.cdc.gov/diabetes/ndep/pdfs/8-road-to-health-barriers-quiz-508.pdf. Accessed August 1, 2011.

[22] Weissman MM, Sholomskas D, Pottenger M, Prusoff BA, Locke BZ Assessing depressive symptoms in five psychiatric populations: a validation study. Am J Epidemiol. 1977;106(3):203-214. doi:10.1093/oxfordjournals.aje.a112455 900119

[23] US Department of Health and Human Services Physical Activity Guidelines for Americans. 2nd ed Wasington, DC: US Department of Health and Human Services; 2018.

[24] Smeets T, Brug J, de Vries H Effects of tailoring health messages on physical activity. Health Educ Res. 2008;23(3):402-413. doi:10.1093/her/cyl101 17032705

[25] Fukuoka Y, Vittinghoff E, Jong SS, Haskell W Innovation to motivation—pilot study of a mobile phone intervention to increase physical activity among sedentary women. Prev Med. 2010;51(3-4):287-289. doi:10.1016/j.ypmed.2010.06.006 20600263 PMC2939294

[26] Fukuoka Y, Lindgren T, Jong S Qualitative exploration of the acceptability of a mobile phone and pedometer-based physical activity program in a diverse sample of sedentary women. Public Health Nurs. 2012;29(3):232-240. doi:10.1111/j.1525-1446.2011.00997.x 22512424 PMC4219361

[27] Bravata DM, Smith-Spangler C, Sundaram V, Using pedometers to increase physical activity and improve health: a systematic review. JAMA. 2007;298(19):2296-2304. doi:10.1001/jama.298.19.2296 18029834

[28] Middelweerd A, Mollee JS, van der Wal CN, Brug J, Te Velde SJ Apps to promote physical activity among adults: a review and content analysis. Int J Behav Nutr Phys Act. 2014;11:97. doi:10.1186/s12966-014-0097-9 25059981 PMC4132213

[29] Knight E, Stuckey MI, Prapavessis H, Petrella RJ Public health guidelines for physical activity: is there an app for that? a review of android and apple app stores. JMIR Mhealth Uhealth. 2015;3(2):e43. doi:10.2196/mhealth.4003 25998158 PMC4456485

[30] Garcia-Ortiz L, Recio-Rodriguez JI, Agudo-Conde C, ; EVIDENT Investigators Group; Mobilizing Minds Research Group Long-term effectiveness of a smartphone app for improving healthy lifestyles in general population in primary care: randomized controlled trial (Evident II study). JMIR Mhealth Uhealth. 2018;6(4):e107. doi:10.2196/mhealth.9218 29702473 PMC5948409

[31] Recio-Rodriguez JI, Agudo-Conde C, Martin-Cantera C, ; EVIDENT Investigators Short-term effectiveness of a mobile phone app for increasing physical activity and adherence to the Mediterranean diet in primary care: a randomized controlled trial (EVIDENT II study). J Med Internet Res. 2016;18(12):e331. doi:10.2196/jmir.6814 27993759 PMC5206481

[32] Pew Research Center Mobile fact sheet. http://www.pewinternet.org/fact-sheet/mobile/. Accessed July 25, 2018.

[33] Mattila E, Lappalainen R, Välkkynen P, Usage and dose response of a mobile acceptance and commitment therapy app: secondary analysis of the intervention arm of a randomized controlled trial. JMIR Mhealth Uhealth. 2016;4(3):e90. doi:10.2196/mhealth.5241 27468653 PMC4981693

[34] Fukuoka Y, Gay C, Haskell W, Arai S, Vittinghoff E Identifying factors associated with dropout during prerandomization run-in period from an mHealth physical activity education study: the mPED trial. JMIR Mhealth Uhealth. 2015;3(2):e34. doi:10.2196/mhealth.3928 25872754 PMC4411363

[35] Pew Research Center. Tech adoption climbs among older adults. https://www.pewinternet.org/2017/05/17/tech-adoption-climbs-among-older-adults/. Accessed September 30, 2018.

[36] Tudor-Locke C, Johnson WD, Katzmarzyk PT Accelerometer-determined steps per day in US adults. Med Sci Sports Exerc. 2009;41(7):1384-1391. doi:10.1249/MSS.0b013e318199885c 19516163

