# Supplementary material for: Short- and Long-term Effects of a Mobile Phone App in Conjunction With Brief In-Person Counseling on Physical Activity Among Physically Inactive Women: The mPED Randomized Clinical Trial
Source: JAMA Netw Open. 2019 May 24;2(5):e194281. doi: 10.1001/jamanetworkopen.2019.4281 (PMC6632135; doi:10.1001/jamanetworkopen.2019.4281)
Supplement: Supplement 1. — Trial Protocol [file jamanetwopen-2-e194281-s001.pdf]

mPED (Motivational Physical Education Program) Trial Protocol

Official Title	Applying Mobile-Persuasive Technologies to Increase Physical Activity in Women
UCSF IRB approval #	No. 10-04566
ClinicalTrials.gov #	NCT01280812

1.0 Brief Summary

Fewer than half of adult women in the US engage in recommended levels of physical activity. [1] As a consequence, they are at increased risk of heart disease, stroke, hypertension, diabetes, certain cancers, and premature mortality. [2] Given the rapid growth of mobile phone technology and the increasing number of users, we developed an interactive mobile phone-based physical activity intervention for physically inactive women. We assessed the feasibility and acceptability of this intervention in a 3-week pre-post study in which we had rapid enrollment, no attrition, high compliance (95% compliance with an Omron HJ-720ITC and 85% compliance with a cell phone diary), and a significantly increased step count.[3] We now propose to conduct a randomized, controlled trial to assess the efficacy of the mobile phone-based physical activity intervention on increasing physical activity over a 3-month period. 192 sedentary women will be randomized in a 1, 1, to -to-1 ratio to a Control, Regular, or Plus group. All participants will receive an Omron Active Style Pro HJA-350IT and a study app and will asked to wear an Omron Active Style Pro HJA-350IT and to send their total number of steps through a mobile phone diary before going to bed each night. Only participants in the intervention groups will receive the physical activity intervention through their mobile phones via daily prompts, weekly video clips, and customized feedback and self-monitoring. The primary and secondary outcomes will be total steps per day and moderate to vigorous physical activity (MVPA) measured by Omron Active Style Pro HJA-350IT. Another secondary outcome is the 7-day physical activity recall questionnaire.

To provide insight into how best to maximize the potential for sustained physical activity after completion of the 3-month program, women in the intervention groups who complete the physical activity program will be further informed of a 6-month maintenance condition. We will also explore the role of potential mediating factors (physical activity barriers, self-efficacy, and social support,) and moderating factors (BMI and age) on changes in physical activity. If proven effective, a major advantage of a mobile phone-based intervention is that it could potentially be administered to a large number of women.

2.0 Background and Rationale

The Centers for Disease Control (CDC) and the American College of Sports Medicine have recommend that adults engage in at least 30 minutes of moderate physical activity on most days (and, preferably, on all days). [2] However, more than 50% of women do not participate in recommended levels of physical activity; approximately 25% of women do not engage any type of physical activity at all. [1]. Physical inactivity is a major risk factor among the top 10 causes of death (e.g., heart disease, cancer, and stroke) in U.S. adults. Thus, the financial burden related to physical inactivity is enormous. In 2000, health care costs associated with physical inactivity topped $76 billion.[4] The CDC estimated that if 10% of adults began a regular walking program, $5.6 billion in heart disease treatment costs could be saved. [4]. Thus, physical inactivity still remains a pressing public health issue in the United States today.[5]. This study is innovative in its use of mobile phones to deliver a tailored, interactive physical activity intervention for physically inactive women. The use of this widely available mobile technology to deliver physical activity interventions to this population could overcome many of the problems with other communication technologies, such as the Internet. First, barriers to mobile phone usage by women are receding; 86% of US adults use mobile phones, and women use mobile phones more than men.[6]. Second, once participants are enrolled, they receive the intervention through their mobile phones, which eliminates the requirement for participants to log onto the Internet or to schedule a telephone appointment with a researcher. Third, by using a daily prompt (text message or video clip format), the intervention can be delivered immediately and without the need for cumbersome text messaging. Fourth, accurate and timely data collection is greatly enhanced with the use of mobile phones because participants can send data directly to researchers (in response to automated prompts), without the need to remember to record data manually each day in a diary, a practice that is prone to recall bias. Fifth, the mobile phone-based physical activity intervention program can be installed on each woman's own mobile phone within a few minutes. Most importantly, patients receive tailored, motivational messages each day that provide positive feedback in an effort to minimize participant attrition over time. Lastly, maintaining physical activity is extremely difficult for sedentary

women; yet, little empirical evidence is available to evaluate critical components of long-term adherence to physical activity. The proposed study compares two low-cost maintenance strategies (providing different levels of self-monitoring and feedback) designed to help women sustain their physical activity.

Maintenance of physical activity: Maintenance is often defined as exercising regularly at least 6 months after cession of the intervention. [7-8]. Maintaining regular physical activity is critical to reach one's full health benefits. Yet, high attrition is an issue, as reported attrition rates can be as high as 84%. However, empirical evidence of effective intervention strategies for maintenance of physical activity is at an early stage. There is an urgent need to establish innovative models of maintenance strategies for physical activity. Thus, we propose to evaluate two low-cost maintenance strategies.

Factors associated with physical activity maintenance: Choice of physical activity format seems to be an important factor. The home-based programs have significantly greater adherence over traditional group exercise programs at one year, as it may simply be too difficult for subjects to sustain group exercise regimen for long periods. Demographics (such as female gender, old age, and high BMI) and psychological factors (such as mental stress and depressive symptoms) are also associated with low physical activity maintenance. [7, 9] In contrast, frequent self-monitoring, goal setting, and frequent contact/feedback with subjects appear to be associated with sustained physical activity. Thus, in the proposed study, we have included more frequent self-monitoring and feedback through a physical activity diary into one of the randomization arms (Omron Active Style Pro HJA-350IT plus mobile phone diary) for the maintenance phase.

3.0 Clinical Trial Aims

Aim 1: To assess the efficacy of the mobile phone-based physical activity intervention (Regular and Plus groups) on increasing physical activity at 3 months compared to the Control group.

Aim 2: To compare the efficacy of the 6-month maintenance intervention (Plus group: Omron Active Style Pro HJA-350IT plus mobile phone diary) to the Regular group (Omron Active Style Pro HJA-350IT only) on physical activity.

Aim 3: To explore the role of potential mediating factors (physical activity self-efficacy, social support, outcome expectation, and decisional balance) and moderating factors (Body Mass Index (BMI) and age) on changes in outcomes at 3, 5, 7 and 9 months.

4.0 Study Design

192 will be randomly assigned in equal proportions to the Control, Regular and Plus groups, using randomly permuted blocks of randomly selected block sizes of 3, 6, and 9. Table 2 shows the different components among the three groups. All groups will receive an Omron Active Style Pro HJA-350IT and will be asked to wear an Omron Active Style Pro HJA-350IT every day for 9 months. However, only women in the Regular and Plus groups will receive a 20-minute in-person physical activity counseling and a study app that includes daily prompts, video clips, and diaries. Furthermore, women in the intervention groups will receive immediate feedback and self-monitoring (a bar graph) immediately after they enter their mobile phone diary before going to bed. After completing the 3-month mobile phone-based physical activity program, women in the Plus group will be asked to continue using the diary on the study app and wearing an Omron Active Style Pro HJA-350IT for additional 6 months. The Regular group will stop using the study app and only keep wearing an Omron Active Style Pro HJA-350IT. The Control will continue wearing an Omron Active Style Pro HJA-350IT.

Figure 1. Study design

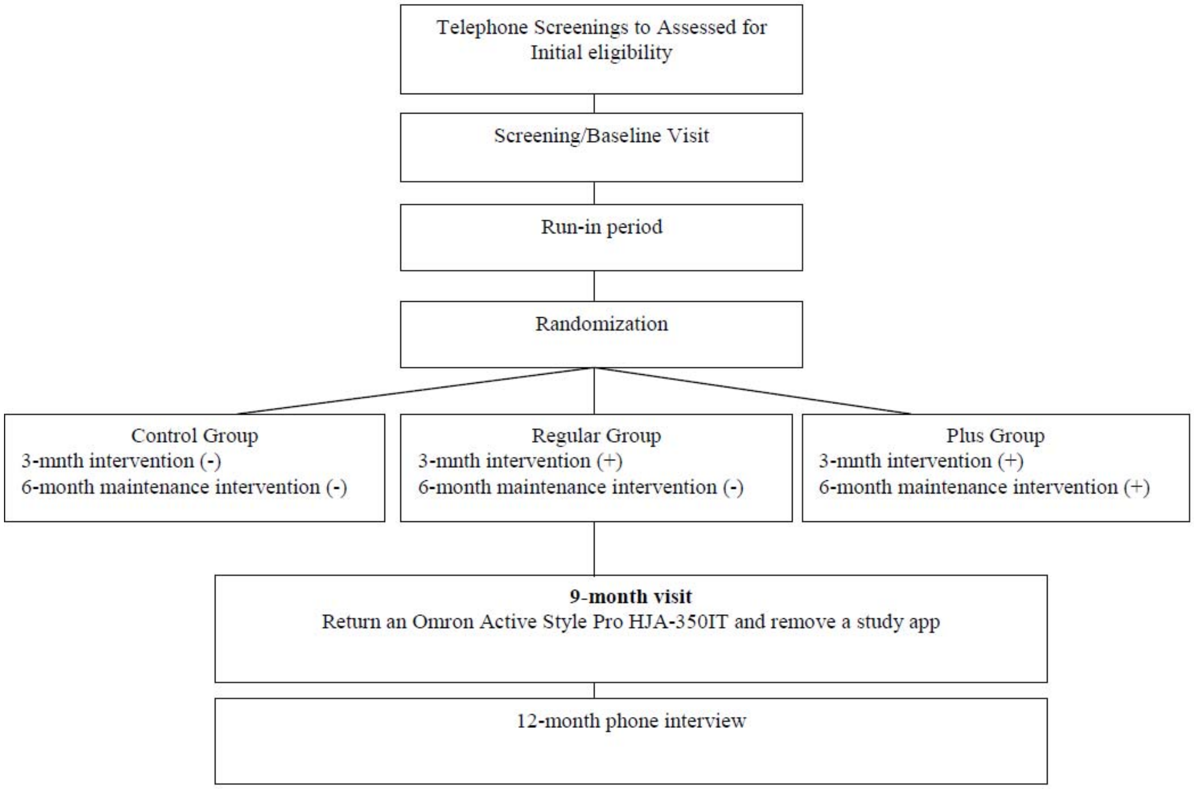

Table 1. Components of the physical activity intervention and control groups

**3-month intervention phase**	**3-month physical activity Intervention Group**		**Control group (Omron Active Style Pro HJA-350IT)**
**1. Omron Active Style Pro HJA-350IT functions (display)**			
1 Daily steps	•		•
**2. Mobile phone daily diary components**			
2a. Diary	•		
2b. Automated feedback and self-monitoring (weekly step count graph and encouraging message)	•		
**3. Intervention components**			
3a. A-20 minute face-to-face intervention at baseline	•		
3b. Daily messaging daily prompt component	•		
3c. Mobile phone video-clip intervention component	•		
**6-month maintenance phase**			
	**Maintenance Intervention-PLUS group**	**Maintenance Intervention-REGULAR Group**	**Control group (Omron Active Style Pro HJA-350IT)**
**1. OmronActive Style Pro HJA-350IT functions (display)**			
1.a. Daily steps	•	•	•
**2. Mobile phone daily diary components**			
2a. Diary	•		
2b. Automated feedback and self-monitoring (weekly step count graph and encouraging message)	•		

3. 5, 7, and 9-month research visits			
3a. A copy of accumulate physical activity performance report	•	•	
3.b. Performance evaluation	•	•	
**4. 12-month Follow-Up**			
4a. Follow-Up phone call	•	•	•

5.0 Study Population

5.1 Sample Size

In order to meet our recruitment goal of 5 to 6 subjects per month, we will consecutively recruit from Bay Area communities in California a total of 192 sedentary women who meet all inclusion criteria to this study.

5.2 Inclusion Criteria

1. Physically inactive at work and/or during leisure time using the Brief Physical Activity Recall questionnaire[52] and based on baseline average daily steps
2. Intend to be physically active
3. Female, age ≥25 to 69
4. Access to a home telephone or a mobile phone
5. Speak and read English
6. Body Mass Index (BMI) between 18.5 - 43.0 kg/m^2

5.3 Exclusion Criteria

1. Known medical conditions or other physical problems that need special attention in an exercise program (e.g., prior myocardial infarction, history of angioplasty, history of angina, admission to the hospital for evaluation of chest pain, use of nitroglycerin to treat angina, uncontrolled hypertension, and diabetes mellitus with insulin treatment).
2. Planning a trip abroad during the study period.
3. Known bone or joint problems that impair the ability to do moderate physical activity (e.g., arthritis, osteoporosis).
4. Pregnant/Delivered a baby during the last 6 months.
5. Known severe hearing or speech problem.
6. History of eating disorders (e.g. binge eating disorder, bulimia nervosa, anorexia nervosa).
7. Currently participating in lifestyle modification programs or research studies that may potentially confound the results of the study.
8. History of bariatric surgery or future plans for bariatric surgery in the next 10 months.

6.0 Ethics

6.1 Institutional Review Board

The study protocol, informed consent form, study questionnaires, educational and recruitment materials must be approved by the Institutional Review Board (IRB). Protocol amendments generated during the study must be approved by the IRB prior to their implementation. Copies of all IRB approval letters will be sent to the Project Office at the NIHLBI. Reports issued by the study Data and Safety Monitoring Board will be distributed to the investigators to be forwarded to the IRB. Any serious adverse events that occur during the trial must be reported to each IRB.

6.2 Informed consent form

Before individuals may participate in any screening procedures, informed consent for all phases of the trial must be obtained. The consent form will explain in lay language the goals of the study, the visits and procedures, and the risks and benefits of participating. Any amendment to the protocol generated during the study that impacts participants will be reflected in a revised consent form that must be signed again by the participant. All participants will receive a copy of the consent form.

7.0 Study Schedule and Procedures

7.1 Summary of Study Visits

Table 2. Study measures and data collection schedule

		Intervention phase			Maintenance phase			
**Study Measurements**	**Baseline/ Screening visit**	**Randomization**	**1.5 month**	**3 month**	**5 month**	**7 month**	**9 month**	**12 month**
**Outcomes**								
1. Omron Active Style Pro HJA-350IT (total steps/day, duration of MVPA/day)		•	•	•	•	•	•	
2. 7-day Physical Activity Recall		•		•			•	•
**Covariates**								
3. Self Efficacy for Physical Activity	•			•			•	
4. Social support	•			•		•	•	
5. Short Form Health Survey (SF-12)		•		•			•	
6. Barriers to Being Active Quiz	•			•			•	
7. Center for Epidemiological Studies Depression Scale	•			•			•	
8. Sociodemographics and medical history	•							
9. Resting blood pressure/waist circumflex	•			•		•	•	
10. Body weight, height, and body mass index	•			•		•	•	
11.Blood test Lipid profile, fasting glucose, and A1C	•					•		
12.Adverse Events Checklist			•	•	•	•	•	
13.Telephone Follow-Up Interview<br>• assess individual's recent physical activity								•

7.2 Randomization Procedures

After a run-in period, we will randomize these women to one of the three groups. We have estimated an attrition rate of 12% at 3 months; thus, we anticipate that at least 112 subjects in the intervention groups and 56 subjects in the control group will complete the study. At the end of the run-in period, participants meeting the compliance requirements will randomly assigned in equal proportions to the Control, Regular and Plus groups, using randomly permuted blocks of randomly selected block sizes of 3, 6, and 9. Since the intervention will not be blinded, block sizes will vary randomly from 3, 6, and 9 in a schedule that is not known to investigators. Using computer algorithms, data management staff will prepare a set of sealed, opaque envelopes. On the outside, the envelopes will be numbered consecutively with the randomization sequence number; on the inside the envelopes will contain study group assignment.

7.3 Study Visits

7.3.1 Recruitment Strategies

Based on our previous recruitment experience and pilot study, Craigslist, the San Francisco Examiner and San Francisco Bay Guardian (free local newspaper), flyers posted at the UCSF campus and community clinics, and flyers posted at supermarkets are effective ways to recruit women from the San Francisco Bay Area. We tested the recruitment methods in our pilot study, in which we were able to successfully recruit minority (approximately 58.5%) and low-income women (32% annual household income < $20,000).

To recruit 192 subjects, we estimate that we need to screen approximately 800 subjects over the telephone. We estimate that we will be able to recruit and randomize at least 5 to 6 women per month. Hence, it will take approximately 34 months to recruit 192 women. We will attempt to minimize obstacles to participation; for example, travel barriers will be

addressed by providing transportation or parking costs, and scheduling barriers will be minimized by offering a flexible assessment schedule.

7.3.2 Telephone Screening

Women who are interested in the study will call or email a research assistant. The research assistant will screen potential participants over the telephone. The research assistant will briefly explain the study. If the participant meets all inclusion criteria, she will be scheduled for a screening visit at the UCSF Laurel Heights office.

7.3.3 Screening/Baseline Visit

During the first study visit to the research office, the research assistant will explain the study using a written consent form. Particular emphasis will be placed on the concepts of run-in period, randomization and daily mobile phone and Omron Active Style Pro HJA-350IT use. If the participant agrees to participate in the study, written consent will be obtained. After obtaining consent for participation in the study, the research assistant will conduct a structured interview using the questionnaires. The research staff will also measure the participant's weight, height, and blood pressure. It takes 45 to 60 minutes to complete the baseline data collection. Immediately after the structured interview, all participants will have hands-on training in using an Omron Active Style Pro HJA-350IT and the daily mobile phone diary with a research assistant.

If the participants have an active chronic disease (e.g diabetes with oral hypoglycemic medication, more than five or equal to five cardiovascular risk factors, etc.) or any medical concern, we will notify the participant's primary care physician of her enrollment (by mail/fax) after the screening/baseline visit. A primary care physician will have at least 14 days to respond, if there are any contradicting medical conditions. The National Guidelines on Physical Activity and Public Health [(10)] do not recommend a cardiovascular evaluation before asymptomatic individuals undertaking a walking program, due to the extremely low incidence of serious adverse events, and low sensitivity with high cost.

7.3.4 Run-in Period

Prior to randomization, we will conduct a run-in period in which women will be given a run-in app and an Omron Active Style Pro HJA-350IT and instructed to wear the Omron Active Style Pro HJA-350IT daily and to complete the daily mobile phone diary. Although we did not have a problem with compliance with these requirements during our 3-week pilot study [(3)] , we are including a run-in period for the following reasons; a run-in period will enable us to screen for non-compliant participants who are not willing to wear an Omron Active Style Pro HJA-350IT daily (i.e., with certain kinds of clothing) or who do not complete the daily mobile phone diary. Only participants who complied at least 80% will be randomized. Second, recorded Omron Active Style Pro HJA-350IT data during the run-in period will be used as baseline daily steps. In order to obtain accurate baseline data, we will display only the time (like a 24-hour clock) on the Omron Active Style Pro HJA-350IT, although the Omron Active Style Pro HJA-350IT will record daily steps and intensity of physical activity during the run-in period. At the end of a day, participants will be asked to report their estimated daily steps on a mobile phone dairy during the run-in period.

7.3.5 Randomization Visit

At the randomization visit, when eligibility has been confirmed by completion of the eligibility checklist, a research assistant will enter the date, participant's name, and study ID (assigned at the screening visit) consecutively in a randomization log, select the next numbered envelope, enter the randomization sequence number listed on the outside of the envelope, then open the envelope and enter the study group assignment contained inside the envelope on the randomization log. All opened and unopened envelopes will be retained at a research office for review during data collection and at the end of the trial. Randomization dates and times should follow the order of the randomization sequence numbers, providing a check on validity. Screening data will be retained by study ID for women not randomized, facilitating studies of the recruitment process. Women assigned to the intervention groups (Regular and Plus groups) will receive an initial 20-minute in-person intervention. Participants will be instructed to avoid mentioning anything regarding their intervention. While participants will not be blinded to their assignment, they will not be informed about the study hypotheses or primary outcome measures.

7.3.6 Follow-up Visits During the Intervention Phase for Intervention and Control Groups

A research staff will schedule follow-up study visits to the study center at 1.5 and 3 months. The week prior to the 1.5 and 3-month dates, a research assistant will email or mail an appointment notification to the participant's home. In addition, the research staff will confirm the appointment over the telephone three days prior to the appointment. Participants will be asked to bring their Omron Active Style Pro HJA-350IT and mobile phone to the study visit. The Omron Active Style Pro HJA-350IT will hold data regarding daily total steps and intensity during the prior 150 days.

7.3.7 Follow-up at 5, 7, 9 and 12 months During the Maintenance Phase

Although the Omron Active Style Pro HJA-350IT can record 150 days of continuous data, the battery needs to be changed approximately once every three months. All participants will be asked to bring their Omron Active Style Pro HJA-350IT to the research office to download their data at 5, 7, and 9 months. We will not introduce any new skills during these follow-up research visits. At 12-months, a follow-up telephone call will be made to all participants to assess individual's recent physical activity, social support, and mood. It will take approximately 15 minutes.

7.4 Study Measures

7.4.1 Primary Outcome

7.4.1.1 Total Steps per Day

We will use the Omron Active Style Pro HJA-350IT (triaxial accelerometer) to measure total steps per day. This comes with a USB Connection and PC Software. The Active Style Pro HJA-350IT can display daily steps. The advantage of using the Omron Active Style Pro HJA-350IT is that the accelerometer will automatically reset the step count every evening at midnight while still allowing participants to view the past 7 days of step counts. Another advantage of using this Omron Active Style Pro HJA-350IT is that a researcher can select 1 of 4 Omron Active Style Pro HJA-350IT screen displays: (1) Steps display only, (2) MET display only, (3) 24-hour clock display (does not show any physical activity information), and (4) Steps, MET, and weekly average activities during the last four weeks. Data from the most recent 150 days performance will also be automatically stored so they can be directly downloaded to a computer. The criterion for acceptable Omron Active Style Pro HJA-350IT data is that the downloaded data from the Omron Active Style Pro HJA-350IT must show that the participant wore the Omron Active Style Pro HJA-350IT equal or greater than 8 hours/day, and equal or greater than 4 days per week.

7.4.2 Secondary outcomes

7.4.2.1 Intensity of Physical Activity per Day

The Active Style Pro HJA-350IT can also measure activity intensity in the last 60 seconds by metabolic equivalent (MET).

7.4.2.2 7-day Physical Activity Recall (PAR)

An interviewer-administered 7-day physical activity recall (PAR) will be used to assess physical activities performed during the week preceding each visit. The PAR is a widely used and well-validated self-report recall instrument that assesses the frequency, duration, and intensity of physical activity. [(11)] It yields several physical activity indexes (minutes of exercise at each level of exercise intensity, number of days exercised, and a rough estimate of caloric expenditure over the week).

The PAR is administered in a semi-structured interview format, with the administrator probing for activity and clarifying the intensity of activity reported. Two-week test-retest reliability has been reported to range from 0.61 to 0.99, and correlations between the PAR and other measures of physical activity have been reported at 0.61 (VO2 max), 0.66–0.83 (self-reported activity), and 0.28–0.43 (accelerometer). [(12)] Total minutes spent in moderate and vigorous activity during the previous week will be used as the subjective physical activity outcome measure.

7.4.3 Other Study Measures

7.4.3.1 Self-Efficacy for Physical Activity Survey

The Self-Efficacy for Physical Activity will be used to measure how confident the participant is to engage in physical activity in a specific situation. A sample item is, "I am confident I can participate in regular physical activity when I am tired." The measure consists of five items on a scale of 1 to 5, "1" being "not at all confident" and "5" being "very confident." The possible range of scores is from 5 to 25 points. Higher scores indicate higher self-efficacy for physical activity. Reported internal consistency ranges from 0.78 to 0.82. [(13)] This measure has been widely used in adult women and men.

7.4.3.2 Social Support for Physical Activity

The Social Support and Exercise Survey will be used to measure both friend and family social support related to physical activity during the past three months. [14] The measure comprises two subscales (friend and family support subscales). Each subscale has 12 items with 5-point Likert scales (ranging from 1, "none" to 5, "very often"). The ratings of all 12 items are summed for a subtotal score. Internal consistency (Cronbach's alpha) of the measure was 0.83 in previous studies. Test-retest reliabilities of the measure have ranged from 0.79 to 0.90 for both scales. Reported internal consistencies ranged between 0.80 to 0.93 for both scales. [13]

7.4.3.3 Short Form Health Survey (SF-12)

The 12-Item Short Form Health Survey (SF-12) was developed for the Medical Outcomes Study (MOS), a multi-year study of patients with chronic conditions. The 12-Item Short-Form Health Survey was developed to describe mental and physical health status of adults and to measure the outcomes of healthcare services. Studies have demonstrated the reliability and validity of the SF-12 to be extremely well correlated with the SF-36. [15]

7.4.3.4 Barriers to Being Active

Barriers for physical activity will be measured by the Barriers to Being Active Quiz. This measure contains seven subscales of the most frequently listed barriers, such as (1) lack of time, (2) social influence, (3) lack of energy, (4) lack of willpower, (5) fear of injury, (6) lack of skill, and (7) lack of resources. Each subscale contains three items. Participants will be asked to select one answer on a scale of 0 to 3, "0" being "very unlikely" and "3" being "very likely." A subtotal score of 5 or above in any subscale shows that this is an important barrier for participants to overcome. The mobile phone intervention group will discuss with a research assistant how to overcome these barriers during the face-to-face session. The Centers for Disease Control and Prevention has posted this questionnaire on their web site to promote physical activity (16).[14]

7.4.3.5 Center for Epidemiological Studies Depression Scale

The Center for Epidemiological Studies Depression Scale (CES-D) [17] consists of 20 items where participants are asked to rate each item on a scale from 0 to 3 in response to the question, "How often have you felt this way during the past week" where 0 = rarely or none of the time (less than 1 day), 1 = some or a little of the time (1–2 days), 2 = occasionally or a moderate amount of time (3–4 days), and 4 = most or all of the time (5–7 days). CES-D scores range from 0 to 60; higher scores indicate more severe depressive symptoms.

7.4.3.6 Sociodemographic Characteristics and Medical History

Through structured interviews a research assistant will collect sociodemographic characteristics (ethnicity, marital status, date of birth, education level, employment status, annual household income, marital status, number living in the home, employment status, and health insurance status). Medical history (current smoking status, alcohol intake) and current medications (name and dosage) will be also collected.

7.4.3.7 Resting Blood Pressure

The Omron Automatic Blood Pressure Monitor with IntelliSense® (HEM-711AC) will be used to measure resting systolic and diastolic blood pressure. Following a rest period of at least five minutes, two blood pressure readings will be taken in each upper arm (at one-minute intervals). We take an average of two measurements in each arm.

7.4.3.8 Body Weight, Height, and Body Mass Index

The Healthometer Professional Floor Scale will be used to measure body weight. The scale reads in kilograms and pounds simultaneously (up to 180 kg or 400 lbs). Body weight is measured to the nearest 0.1 kg (0.25 lb). Participants will be asked to wear a cloth hospital gown, without shoes or socks. Height will be measured by the Healthometer PORTROD Height Rod with increments of 1 mm (1/16 in). Body mass index will be determined by dividing the body weight (kg) by the square of the height (m2).

7.4.3.9 Waist and Hip Circumferences

Waist circumference will be measured at a level midway between the lower rib margin and iliac crest with the tape all around the body in horizontal position.

7.4.3.10 Blood Analyses

A blood specimen collected at the baseline and at the 7 month follow-up will include the following tests: hemoglobin A1c, blood glucose, total cholesterol, HDL cholesterol, LDL cholesterol, and triglycerides. The specimen requirements for Quest Diagnostics are the following:

- A1c (39157): 2 mL whole blood
- Glucose (10160): 1 mL serum in a Tiger top tube
- Cholesterol Panel (which includes total, HDL, LDL and TriG): 1 mL serum in a Tiger top tube

This will result in the collection of 2 tubes of blood:

- 1 Tiger top tube of 2 mL
- 1 Lavender top tube of 2 mL whole blood

If the subject wishes to receive the results of her blood test, the results will be given at the end of her study participation.

8.0 Physical activity interventions [(3)]

There are two components to the physical activity intervention: 1) brief in-person physical activity session and 2) the mobile phone application (app) along with an Omron Active Style Pro HJA-350IT triaxial accelerometer. All women randomized into the PLUS and REGULAR groups will receive them for the 3 months in an identical fashion.

Brief in-person intervention

Women in the Plus and Regular groups will be given a brief in-person interactive session by trained research staff. The in-person intervention is a structured interview containing seven domains: 1) overview of the physical activity program and tailored short and long-term goal setting; 2) education about duration and intensity of brisk walking and the health benefits of exercise; 3) identification of barriers to increasing physical activity and development of strategies to overcome these barriers; 4) value and identification of social sup- port while increasing physical activity; 5) relapse prevention; 6) education about healthy diet and weight maintenance; and 7) physical activity safety. The in-person intervention is designed to be interactive and actively elicits the participant’s participation. An individualized written physical activity plan will be developed during the face-to-face intervention. The trained research staff will revisit and revise the plan at the 1.5- and 3-month visits.

Apps

Two different mobile phone applications were created for this RCT: a content neutral run-in application and the trial application (the physical activity intervention). The trial app will have three components 1) the daily message/video clip, 2) daily mobile phone diary, and 3) other functions ("Talk to us”, “Summary”, and “Help” menus). The run- in application will mimic the format of the trial application, but will not contain any content to support a physical activity program. The run-in and trial apps were developed to run on two different mobile phone platforms: the Java 2 platform, Micro Edition (J2ME) and the iOS (iPhone) platform. If the participant has a compatible mobile phone or an iPhone, the application is installed on their personal phone. If participants do not want to install the application on their phone or do not have a compatible mobile phone, we provide a Motorola RAZR v3xx or a Pantech LASER study mobile phone, along with voice, text messaging (short message service) and data plans.

<u>Daily messages/video clips</u>

The daily messages/video clips of the trial application reinforce the seven domains addressed in the brief in-person session; the domains are divided into twelve weekly themes. A preprogrammed daily message or video clip will be automatically sent at a predetermined time between 11 a.m. to 3p.m. The daily message will be accessible on the mobile phone until 7 p.m., if no reply is made by this time, the daily message will disappear from the phone. An auto- mated text message is generated to alert participants that the daily prompt has arrived. Each daily prompt begins with a message from the research staff, followed by a question relevant to the message. For example, on Day 5 of Week 4, subjects receive the following daily message: ’Have you let everyone around you know that you are trying to become more active so they can help you meet your goal?’ “No” or “Yes” is selected by pushing the keypad. If “No” is selected, the next screen will display “Let others know your physical activity goal.” If “Yes” is selected, the next screen will display “Nice work!” It takes only 1 one to two minutes to complete the daily prompt each day.

<u>Physical Activity diary</u>

In our three week pilot study, the average timing of mobile diary use was approximately 9:30 pm, but some subjects used the mobile diary even before 8 p.m. In this RCT, the physical activity diary will be accessible after 7 p.m. If no entry is made by 8:30 p.m., an automated text message is generated as a reminder for the participant to record the total number of steps per day and the types, and duration of physical activities. The mobile phone diary program was designed so subjects have to answer questions sequentially. For example, when "Diary" is selected, the first question is: "Did you wear a Omron Active Style Pro HJA-350IT triaxial accelerometer all day today, except for showering, swimming, or sleeping?" If the answer is "Yes," the woman will be directed to enter the number of steps taken that day. To increase accuracy of data entry, a range of steps (1000 and 35,000) was programmed in advance. The participant will immediately receive a daily step histogram showing the daily step count, enabling her to monitor/visualize her progress. If the answer is "No" (i.e. - she didn't wear the Omron Active Style Pro HJA-350IT triaxial accelerometer), she will be asked to select the reason why and will receive suggestions based on her answer. The next question is, "Did you do more than 10 minutes of physical activity since midnight last night?" If "Yes", she will be prompted to select the type and to enter the total number of minutes of physical activity. This entry generates a message providing immediate feedback based on the total number of minutes entered. For example, thirty minutes of physical activity generates the response, "Excellent job!"

<u>Other functions</u>

In addition to daily message (intervention) and mobile phone diary, the trial application includes "Summary", "Help", "Talk to us", and "Weekly goals" menu options. The "Summary" menu includes the material provided in the face-to-face intervention, the "Help" tab lists the research office contact information, and the "Talk to us" function allows the subject to directly send a text message to researchers from the application. The activity goal is automatically updated each week and displayed in the "Weekly goals" tab on the application home screen. The mobile phone trial application Physical activity maintenance.

Maintenance

There are two different 6-month maintenance interventions, Regular (Omron Active Style Pro HJA-350IT triaxial accelerometer only) and Plus (Omron Active Style Pro HJA-350IT triaxial accelerometer plus mobile phone physical activity diary). A 6- month maintenance period follows the 3-month mobile phone and Omron Active Style Pro HJA-350IT triaxial accelerometer based physical activity intervention; The maintenance intervention-REGULAR group will use the Omron Active Style Pro HJA-350IT triaxial accelerometer daily and the maintenance intervention (Plus) will use the Omron Active Style Pro HJA-350IT triaxial accelerometer and physical activity mobile phone diary daily. The purpose of the two different maintenance intervention groups is to gain insight into the "dose-response" to self-monitoring and feedback. In other words, is keeping a mobile phone diary and Omron Active Style Pro HJA-350IT triaxial accelerometer for 6-months a better physical activity maintenance method than a Omron Active Style Pro HJA-350IT triaxial accelerometer alone? The subjects in the control group continue using an Omron Active Style Pro HJA-350IT triaxial accelerometer for these six months of the study. The participants in all three groups will be asked to bring back the Omron Active Style Pro HJA-350IT triaxial accelerometer to a research office to download the Omron Active Style Pro HJA-350IT triaxial accelerometer data at 5, 7 and 9 months. At the 9-month visit, participants will return all Omron Active Style Pro HJA-350IT triaxial accelerometers and study mobile phones (if there is any) to the research office. All participants will receive a follow-up telephone call at 12 months to assess their level of physical activity using the 7-Day Physical Activity Recall Questionnaire.

Control (Omron Active Style Pro HJA-350IT triaxial accelerometer only group)

Participants randomized to the Control group will continue using the Omron Active Style Pro HJA-350IT triaxial accelerometer without the intervention. Following the randomization visit, the Omron Active Style Pro HJA-350IT triaxial accelerometer settings will be changed so that the total steps are visible. Although providing a Omron Active Style Pro HJA-350IT triaxial accelerometer for the control group may temporally increase physical activity from baseline, we expect physical activity will not continue to increase in this group after the first few weeks of the study.

9.0 Data Management

9.1 Data Collection & Processing

9.1.1 Data Collection Plan and Flow Chart: Data will be collected from participants by research staff and through three modes of collection (in-person, Omron Active Style Pro HJA-350IT, and mobile phone). All questionnaire data instruments will be collected via Teleforms and faxed to UCSF San Francisco Coordinating Center. Omron Active Style Pro HJA-350IT data will be downloaded at the research office and pushed to UCSF San Francisco Coordinating Center

server. Mobile phone data will be collected and stored two ways – saved on the mobile phone memory card and transmitted in real-time with directly from the mobile phone to the secure server at the UCSF San Francisco Coordinating Center.

9.1.2 Fidelity of the intervention: After the research assistant has been trained, the PI will observe the first two face-to-face interventions. The research assistant will be also asked to digitally record all in-person interventions. After the intervention, the digitally recorded face-to-face intervention will be downloaded to a password protected research computer. The PI will randomly select and listen to one digitally recorded intervention every two weeks to confirm the fidelity of the intervention. In addition, the length of the in-person intervention and level of women's engagement during the session will be recorded by the research staff.

9.1.3 Data management and quality: We will use the University of California, San Francisco Coordinating Center Data Management Services based on Cardiff Teleform software system. In the beginning of the study, the UCSF Coordinating Center will transfer all questionnaires to machine-readable data forms and set up a password protected research website. All forms, protocol, meeting schedules will be listed in the study website. Once the study begins, a research assistant will transmit the data (e.g. questionnaires) using a standard fax machine immediately after the baseline and follow-up data collection. Submitted data forms are received by an automated fax server that uses OCR technology to acquire the data, which are then "verified" on screen by a data manager and stored in the study database on a Microsoft SQL Server. Each night, all study data are checked for completeness, consistency and invalid ranges. The results are posted to the study website as data queries to be addressed by research staff the following day.

For data analysis, conversion of SQL to SAS/STATA/SPSS is handled by a standard procedure so that data are analysis-ready as soon as they are entered and verified. The major advantages of using this data management service are as follows; 1) eliminate manual data entry, 2) eliminate delays in identifying missing data, 3) eliminate delays in cleaning data (real-time data cleaning); 4) provide current and cumulative recruitment statistics, compliance to the visit schedule, a list of rejected forms/data; and NIH quarterly report. We encourage reviewers to view a demo website at http://www.epibiostat.ucsf.edu/general/it/rds.html to see how we will manage our study data if the study is funded. We will be able to list an operation manual, recruitment reports, and questionnaires. In addition, all diary data transmitted through mobile phone by participants will be also automatically time-stamped and stored in the study website.

9.1.4 Data security: Both mobile phone and web-portal data storage will be Health Insurance Portability and Accountability Act (HIPAA) compliant and will not contain any personal identifier information. Each patient will be assigned a study ID immediately after the study enrollment and this ID will be used to identify the subject. In addition, all data entered by subjects on mobile phones will be uploaded to an automatically password protected server (HIPPA compliant data center). All transmission will be encrypted. All data entries will be stored and time-stamped on the mobile phone if a wireless network is unavailable. Each mobile phone will be password protected. A researcher will program the automatic subject activation and inactivation. As a part of UCSF regulation, encryption software will be installed to all computers and portable backup hard drive to ensure data security and protection of electronic information resources.

10.0 Statistical Analysis, Sample Size and Power Estimates

10.1 General Analysis Considerations

Overview: The primary analyses will be by intention-to-treat, without regard to adherence to intervention protocols. We will use linear mixed models to estimate and compare trends in study outcomes. Outcomes will be transformed as necessary to meet assumptions of normality and equal variance; we note that in the pilot study no transformation was necessary for total steps, the primary outcome.

Primary and secondary outcomes. The primary outcome for Aims 1 and 2 will be total steps per day (as measured by the Omron Active Style Pro HJA-350IT). Secondary outcomes will be the MVPA measured by the Omron Active Style Pro HJA-350IT and self-reported 7-day physical activity recall. Differences in the maintenance period will be tested only if the intervention is shown to be successful during the trial, thereby avoiding inflation for type-I error for the primary endpoint. All other endpoints, including number of aerobic steps and physical activity by self-report, will be regarded as secondary. Over-interpretation of isolated or implausible findings of nominal statistical significance will be avoided by examination of corresponding estimates and confidence intervals and of related results while taking account of the scientific context and relationships among analyses, rather than by formal multiple comparisons adjustments.

Potential complications due to dropout: Our pilot data leads us to expect good retention and reliable reporting of outcome variables, and we will make wide ranging efforts to promote continued participation and reporting of outcomes as well as adherence to the intervention. The proposed linear mixed models work with unbalanced data and are known to give consistent estimates of model parameters under the assumption that data are missing at random (MAR), given treatment assignment, time, and the outcomes that are observed. If dropout is substantial (more than 20% at 3 months or 40% at 9 months), we will first adjust for baseline correlates of retention, making the MAR assumption less stringent. In addition, sensitivity analyses will be conducted in which we multiply imput missing outcome data under the conservative assumption that exercise levels fall back to their baseline levels after study dropout. The procedure can be implemented using standard multiple imputation tools in SAS, and was used in the recent PRIDE trial (19).[27]

Assessment of randomization: The validity of the randomization will be assessed by comparing randomized groups on characteristics measured before randomization, using chi-square, Fisher's exact, t- and non-parametric rank tests, as appropriate. If potentially confounding imbalances are found, we will adjust the between-group analyses for potential confounders of treatment assignment.

10.2 Statistical Methods

10.2.1 Specific Aim #1

<u>Aim 1</u>**: To assess the efficacy of the mobile phone-based physical activity intervention on increasing physical activity at 3 months compared to the control group.** In the primary model, we will compare linear time trends in the mean levels of the primary and secondary outcomes, under the hypothesis that the intervention will have a gradually increasing effect; this is motivated by the increasing number of steps participants are asked to take each week and preliminary results from the pilot study. However, verifying this assumption will be important, and alternative models will be considered in secondary analyses if the primary model does not adequately represent the data. To estimate effects of the intervention on total steps per day (as measured by the Omron Active Style Pro HJA-512 350IT), the MVPA minutes per day (also measured by the Omron Active Style Pro HJA-512 350IT), and the 7-day Physical Activity Recall questionnaire scores, we will analyze use analogous linear mixed models, after normalization of the scores as necessary.

10.2.2 Specific Aim #2

<u>Aim 2</u>**: To compare the efficacy of the 6-month maintenance intervention-PLUS (Omron Active Style Pro HJA-350IT plus mobile phone diary) to the maintenance intervention-REGULAR (Omron Active Style Pro HJA-350IT only) on physical activity.** The linear mixed models proposed for Aim 1 will also be used to compare trends in the primary and secondary outcomes during the maintenance period. Adjustment and multiple imputation will again be used in sensitivity analyses assessing the potential influence of dropout. During the maintenance phase, it is often difficult for researchers to determine when participants relapse or withdraw from physical activity. However, in our study, we will be able to determine when participants returned to baseline physical activity (stop exercising) and when participants stopped wearing an Omron Active Style Pro HJA-350IT (defined as ≥ than 7-day consecutive missing data) because our Omron Active Style Pro HJA-350IT can record 150 continuous days of activity. Even information as to when women stop wearing an Omron Active Style Pro HJA-350IT will aid in understanding maintenance strategies.

<u>**Sample size and power for Aims 1 and 2.**</u> We assumed that the net effect of the intervention would be to increase average daily steps from 5200 to 8800 in the intervention group, and from 5200 to 6600 in controls, for a net effect of 2200 steps. These assumptions are based on our pilot data and recently published review papers (20, 21).[26, 28] During the maintenance period, we assumed that the difference between Plus and Regular groups in average daily steps at 9 months, net of any difference occurring by chance at the start of the maintenance period, would be 1100 steps. In both periods, we used data from WIN to estimate the standard deviation of the averaged daily steps as 3000. We also used data from WIN to estimate that the correlation between monthly average values of daily steps would decline from 0.88 between sequential months to approximately 0.82 at visits 3 months apart. Finally, we assumed that 88% of participants would remain in follow-up at 3 months and that 75% would remain in follow-up at 9 months.

Using methods described below, we calculated that a larger sample size will be needed for the maintenance period as compared to the trial. To provide 90% power to detect a between-group difference of 1100 steps per day in the maintenance period, we will require that 114 women (57 each group) in the Plus and Regular groups. On this basis, the implied sample size for the trial is 192 this follows from inflation first by 50%, to account for the fact that the control group will not participate in the extension, and then by an additional factor of 13.6% (=1/.88) to account for expected loss

of 12% of women in the intervention arm during the trial. The sample size of 192 for the trial will provide more than 95% power to detect a difference of 2200 steps, and more than 90% power for net differences as small as 1000 steps.

For secondary endpoints, minimum detectable effects will be reasonably small. For daily steps, with SD of 3000 steps (as estimated from the pilot study), we will have 80% power to detect a net increase in the intervention group of 665 steps during the trial and a net decrease in the regular group of 770 steps during the maintenance period; for this calculation, we assumed that the within subject correlation would be the same as for total steps. For the 7 day physical activity recall questionnaire scores, with SD of 2.0, we will have 80% power to detect between group net differences of 1.22 points in the trial and 1.34 points during the extension; the relatively large minimum detectable standardized effects for this endpoint are driven by the low level of within-subject correlation of only 0.18 observed in the pilot study.

To estimate the sample size, we approximated the variance-covariance matrix S of coefficient estimates to be obtained using the repeated measures regression model for trends in the average daily number of steps, by month, in the intervention and control groups. The matrix S depends on the predictor matrix Xi for each individual, with columns for the intercept, treatment, time, and the time-by-treatment interaction and a row for each available observation; participants who drop out early will have fewer rows. S also depends on W, the inverse of the within-subject correlation matrix of the outcomes. To approximate W, we modeled within-subject correlation as stationary with exponential decline in the lag between repeated measures, with parameter estimates drawn both from the pilot data and from WIN. S was then estimated by the inverse of the sum over participants of the matrix Xi'WiXi, providing estimates of the standard errors of the time-by-treatment regression coefficient, which captures the divergence in trends. Since a regression coefficient estimate is asymptotically normal, it is straightforward to solve a standard formula for power in Wald tests with a normally-distributed test statistic to obtain the sample size required to provide 90% power in 2-sided tests with a type-I error of 5%. The formula can also be solved to obtain minimum detectable effects. This procedure was implemented in R and will be used for both the trial and the maintenance period.

10.2.3 Specific Aim #3

Aim 3: To explore the role of potential mediating factors (physical activity self-efficacy, outcome expectation, and decisional balance) and moderating factors (BMI and age) on changes in outcomes at 3, 5, 7 and 9 months. To assess mediation, we will use the following three steps: Step 1: Estimate the independent association of treatment with the proposed mediator; Step 2: Estimate the independent association of the mediator with subsequent changes in primary and secondary endpoints; and Step 3: Examine attenuation of the estimated effects of treatment when a mediator is added to a model for the independent effect of treatment on the endpoint. To obtain unbiased estimates of the direct effects of treatment not explained by the proposed mechanism, it will be important to control for confounders of the mediating variables. [(22)] Finally, to assess moderation of the treatment effect by age and BMI, we will assess evidence for interaction between treatment assignment and these baseline covariates.

Minimum detectable effects for Aim 3. Recent work by Dr. Vittinghoff [(23)] has shown that if treatment can be shown to affect the mediator (Step 1 above), then showing that the mediator independently affects the outcome, controlling for treatment (Step 2) is equivalent to showing mediation directly (Step 3). Using this approach, we calculate we will have 80% power to show an independent effect of a binary mediator on net change in the primary outcome of 1000 to 2000 steps under a broad range of assumptions about the prevalence of the mediator and the strength of the effect of treatment on the mediator. Since our proposed mediators are continuous, we infer that minimum detectable effects will be smaller. Similarly, we estimate that for binary variables defined in terms of the proposed moderators age and BMI, we will have 80% power to detect differences in the net effect of treatment of 2000 to 3300 steps across the two levels of the moderator, depending on its prevalence. Again, minimum detectable interactions with continuous versions of the moderators should be smaller.

11.0 Reporting of Adverse Events and DSMB

11.1 Plans for Assuring Compliance with Requirements Regarding the Reporting of Adverse Events.

The PI is responsible for reporting adverse events to the research team, to the DSMB, and to the UCSF Committee on Human Research. Mild adverse events will be reported within 10 working days of the occurrence; moderate, severe, life-threatening or fatal adverse events will be reported within 48 hours of the occurrence. This means that research assistants have been trained to recognize, respond to, and record adverse events when they occur or immediately after they occur to insure the safety of the human subjects; and to report adverse events to the PI in a timely manner to insure compliance

with institutional policies on human subject protection. This also means that research assistants engaged in data collection are able to contact the PI as soon as an adverse event occurs.

Specifically, the PI will report the following information in writing to the DSMB and IRB: 1) all serious adverse events associated with the study procedures, and/or 2) any incidents or problems involving the conduct of the study or patient participation, including problems with the recruitment and/or consent processes. The Principal Investigator will provide a discussion of any side effects or problems noticed during each year in the course of the study to the CHR on an annual basis.

11.2 Serious Adverse Events

A Data and Safety Monitoring Plan is required for the proposed project since the study involves a clinical trial of a behavioral intervention. The Data and Safety Monitoring Plan for the proposed project, incorporates the policies on human subject data and safety monitoring specified by the UCSF Committee on Human Research (the UCSF Institutional Review Board).

1. Risk Assessment – Mild Risk

   This study represents a relatively mild risk to study participants since it is a behavioral intervention study.

2. Description of Adverse Event Grading and Anticipated Adverse Events

   An adverse event (AE) is here defined as any unfavorable and unintended sign, symptom, injury or disease temporarily associated with an intervention or procedure, regardless of whether it is considered related to an intervention or procedure that occurs during the course of the study. AE will be scored as follows:

   0 = No adverse event or within normal limits

   1 = Mild AE – did not require treatment

   2 = Moderate AE – resolved with treatment

   3 = Severe AE – resulted in inability to carry on normal activities and required professional medical attention, requires or prolongs hospitalization

   4 = Life-threatening or disabling AE - results in an immediate risk of death and/or results in persistent or significant disability

   5 = Fatal AE

   In this study, we do not anticipate moderate, severe, life-threatening or fatal AEs. We have described anticipated AEs as:

- Participants in the intervention groups will be asked to gradually increase their physical activity (in particular, brisk walking). A potential risk of the study is that some participants may experience musculoskeletal fatigue/pain or injury when they increase physical activity. It is anticipated that this effect will be minimal based on previous work by the investigators. Therefore, the seriousness of this risk is small.
- Confidentiality: Participation in research can involve loss of privacy; however, the participant's names will not be included on questionnaires or other forms, they will be replaced by an identification number.

The PI is responsible for evaluating each AE as it occurs and for notifying the project's Data and Safety Monitoring Committee and the UCSF Committee on Human Research of the occurrence of an adverse event.

11.3 Data and Safety Monitoring Board (DSMB)

The initial DSMB meeting will be held prior to subject recruitment to review the protocol and develop guidelines for DSMB activities and stopping rules. The DSMB members will meet 7 months after recruitment begins, then approximately annually to review study progress, oversee participant safety, monitor data quality, and provide operational and technical advice to the NHLBI project officer and the PI. The DSMB will be comprised of three members with expertise in the operational and biostatisticial aspects of clinical trials, physical activity, health promotion, and women's

health. No member of the DSMB will participate in the study as an investigator or be involved in any way in the conduct of the study.

REFERENCES

1. Centers for Disease Control and Prevention (CDC). Prevalence of regular physical activity among adults--United States, 2001 and 2005. MMWR Morb Mortal Wkly Rep 2007;56(46):1209-12.
2. U.S. Department of Health and Human Services. Physical activity and health: A report of the surgeon general In. Atlanta GA: U.S. Department of Health and Human Services, Center for Disease Control and Prevention, National Center for Chronic Disease Prevention and Health Promotion. ; 1996.
3. Fukuoka Y, Vittinghoff E, Jong SS, Haskell W. Innovation to motivation--pilot study of a mobile phone intervention to increase physical activity among sedentary women. Prev Med. 2010 Sep-Oct; 51(3-4):287-9. PMID: 20600263; PMCID: PMC2939294.
4. Center for Disease and Prevention Control. Preventing obesity and chronic diseases through good nutrition and physical activity. Accessed January 28 2008.
5. Haskell WL, Lee IM, Pate RR, et al. Physical activity and public health: updated recommendation for adults from the American College of Sports Medicine and the American Heart Association. Med Sci Sports Exerc 2007;39(8):1423-34.
6. CTIA - the International Association for the Wireless Telecommnuications Industry. Wireless Quick Facts. Available at: http://www.ctia.org/advocacy/research/index.cfm/AID/10323. Accessed 05-13-2008.
7. Marcus BH, Dubbert PM, Forsyth LH, et al. Physical activity behavior change: issues in adoption and maintenance. Health Psychol 2000;19(1 Suppl):32-41.
8. Bock BC, Marcus BH, Pinto BM, Forsyth LH. Maintenance of physical activity following an individualized motivationally tailored intervention. Ann Behav Med 2001;23(2):79-87.
9. Williams DM, Lewis BA, Dunsiger S, et al. Comparing psychosocial predictors of physical activity adoption and maintenance. Ann Behav Med 2008;36(2):186-94.
10. Haskell WL, Lee IM, Pate RR, et al. Physical activity and public health: updated recommendation for adults from the American College of Sports Medicine and the American Heart Association. Circulation 2007;116(9):1081-93.
11. Sallis JF, Haskell WL, Wood PD, Fortmann SP, Rogers T, Blair SN, Paffenbarger R. (1985). Physical activity assessment methodology in the Five City Project. American Journal of Epidemiology, 121, 91-106.
12. Sallis JF, Buono MJ, Roby JJ, Micale FG, and Nelson JA. (1993). Seven-day recall and other physical activity self-reports in children and adolescents. Medicine and Science in Sports and Exercise; 25, 99-108.
13. Marcus BH, Selby VC, Niaura RS, Rossi JS. Self-efficacy and the stages of exercise behavior change. Res Q Exerc Sport 1992;63(1):60-6.
14. Sallis JF, Grossman RM, Pinski RB, Patterson TL, Nader PR. The development of scales to measure social support for diet and exercise behaviors. Prev Med 1987;16(6):825-36.
15. Sallis JF, Hovell MF, Hofstetter CR. Predictors of adoption and maintenance of vigorous physical activity in men and women. Prev Med 1992;21(2):237-51.
16. Ware J, Jr., Kosinski M, Keller SD. A 12-Item Short-Form Health Survey: construction of scales and preliminary tests of reliability and validity. Med Care 1996;34(3):220-33.
17. Centers for Disease Control and Prevention (CDC). Physical Activity for Everyone. Available at: http://www.cdc.gov/nccdphp/dnpa/physical/everyone/health/index.htm. Accessed 05-01-2008.
18. Weissman MM, Sholomskas D, Pottenger M, Prusoff BA, Locke BZ. Assessing depressive symptoms in five psychiatric populations: a validation study. Am J Epidemiol 1977;106(3):203-14.
19.Subak LL, Wing R, West DS, et al. Weight loss to treat urinary incontinence in overweight and obese women. N Engl J Med 2009;360(5):481-90.
20.Bravata DM, Smith-Spangler C, Sundaram V, et al. Using pedometers to increase physical activity and improve health: a systematic review. JAMA 2007;298(19):2296-304.
21.Tudor-Locke C, Hart TL, Washington TL. Expected values for pedometer-determined physical activity in older populations. Int J Behav Nutr Phys Act 2009;6:59.
22.Cole TJ, Kryakin YV. Sympercents: symmetric percentage differences on the 100 log(e) scale simplify the presentation of log transformed data. Stat Med 2002;21(15):2287-90.
23.Vittinghoff E, Sen S, McCulloch CE. Sample size calculations for evaluating mediation. Stat Med 2009;28(4):541-57.
