# Supplementary material for: Short- and Long-term Effects of a Mobile Phone App in Conjunction With Brief In-Person Counseling on Physical Activity Among Physically Inactive Women: The mPED Randomized Clinical Trial
Source: JAMA Netw Open. 2019 May 24;2(5):e194281. doi: 10.1001/jamanetworkopen.2019.4281 (PMC6632135; doi:10.1001/jamanetworkopen.2019.4281)
Supplement: Supplement 2. — eTable 1. Subgroup Analyses of Age and Body Mass Index (BMI) in Daily Total Steps and Moderate to Vigorous Physical Activity (MVPA) eTable 2. Other Self-Reported Measures eTable 3. Safety Data Summary [file jamanetwopen-2-e194281-s002.pdf]

Supplementary Online Content

Fukuoka Y, Haskell W, Lin F, Vittinghoff E. Short- and long-term effects of a mobile phone app in conjunction with brief in-person counseling on physical activity among physically inactive women: the mPED randomized clinical trial. *JAMA Netw Open*. 2019;2(5):e194281. doi:10.1001/jamanetworkopen.2019.4281

eTable 1. Subgroup Analyses of Age and Body Mass Index (BMI) in Daily Total Steps and Moderate to Vigorous Physical Activity (MVPA)

eTable 2. Other Self-Reported Measures

eTable 3. Safety Data Summary

This supplementary material has been provided by the authors to give readers additional information about their work.

© 2019 Fukuoka Y et al. *JAMA Network Open.*

eTable 1. Subgroup analyses of age and body mass index (BMI) in daily total steps and moderate to vigorous physical activity (MVPA)

	**3 months**				**9 months**			
	**Total steps per day**	**Interaction P value**	**MVPA (minutes) per day**	**Interaction P value**	**Total steps per day**	**Interaction P value**	**MVPA (minutes) per day**	**Interaction P value**
Subgroup	Mean (95% CI)		Mean (95% CI)		Mean (95% CI)		Mean (95% CI)	
Age ≥ 55 years old	2033.5 (933.5 to 3073.6)	.98	18.2 (8.4 to 28.0)	.96	1449.9 (538.0 to 2361.8)	.78	11.4 (2.6 to 20.1)	.36
Age < 55 years old	2055.8 (945.2 to 3166.5)		17.8 (7.3 to 28.3)		1256.5 (281.8 to 2231.2)		5.3 (-4.0 to 14.7)	
BMI ≥30 kg/m^2	2140.5 (1053 to 3227.9)	.89	19.4 (9.1 to 29.7)	.79	1040.5 (95.1 to 1985.8)	.38	8.6 (-0.5 to 17.8)	.92
BMI < 30 kg/m^2	2029.0 (962.4 to 3095.6)		17.3 (7.2 to 27.4)		1638.9((707.9 to 2569.8)		8.0 (-1.1 to 17.0)	

© 2019 Fukuoka Y et al. *JAMA Network Open.*

eTable 2. Other Self-Reported Measures						
		**Baseline**	**3 months**		**9 months** [b]	
**Self-report measures**	**Groups**	**Mean (SD)** [a]	**Difference (95%CI)** [b]	***P* value**	**Difference (95%CI)** [c]	***P* value**
Barriers to being active	Control	12.3 (5.4)	-	-	-	-
	Regular	13.7 (6.4)	-	-	-	-
	Plus	13.4 (5.3)	-	-	-	-
	Intervention vs Control	-	-2.11 (-3.56 to -0.66)	.005	-1.63 (-3.17 to -0.08)	.04
	Regular vs Plus	-	-	-	-0.37 (-2.20 to 1.46)	.69
Social Support	Control	32.0 (9.2)	-	-	-	-
Family	Regular	31.5 (10.9)	-	-	-	-
	Plus	32.8 (9.2)	-	-	-	-
	Intervention vs Control	-	3.49 (1.27 to 5.71)	.002	-0.92 (-2.94 to 1.09)	.37
	Regular vs Plus	-	-	-	0.13 (-2.20 to 2.47)	.91
Friends	Control	31.1 (8.9)	-	-	-	-
	Regular	31.5 (8.2)	-	-	-	-
	Plus	31.8 (8.1)	-	-	-	-
	Intervention vs Control	-	3.45 (1.51 to 5.40)	.001	1.00 (-1.09 to 3.09)	.35
	Regular vs Plus	-	-	-	1.08 (-1.46 to 3.61)	.40
Self-efficacy	Control	18.8 (3.9)	-	-	-	-
	Regular	19.8 (5.1)	-	-	-	-
	Plus	19.0 (4.8)	-	-	-	-
	Intervention vs Control	-	-1.34 (-2.67 to 0.00)	.05	-1.22 (-2.49 to 0.05)	.06
	Regular vs Plus	-	-	-	0.7 (-0.79 to 2.18)	.35
CES-D	Control	9.3 (7.1)	-	-	-	-
	Regular	8.8 (7.0)	-	-	-	-
	Plus	11.1 (8.5)	-	-	-	-
	Intervention vs Control	-	-2.87 (-4.94 to -0.8)	.007	2.39 (-0.05 to 4.82)	.06
	Regular vs Plus	-	-	-	-1.85 (-4.85 to 1.14)	.22
SF-12	Control	47.5 (9.0)	-	-	-	-
Mental component score	Regular	49.0 (8.8)	-	-	-	-
	Plus	49.1 (9.2)	-	-	-	-
	Intervention vs Control	-	1.89 (-0.54 to 4.31)	.13	-1.08(-3.79 to 1.62)	.43
	Regular vs Plus	-	-	-	-0.32 (-3.5 to 2.85)	.84
Physical component score	Control	51.5 (6.8)	-	-	-	-
	Regular	52.1 (5.6)	-	-	-	-
	Plus	50.9 (6.1)	-	-	-	-
	Intervention vs Control	-	-2.24 (-4.34 to -0.15)	.04	-0.56 (-2.65 to 1.53)	.60
	Regular vs Plus	-	-	-	-0.57 (-2.97 to 1.84)	.64

Abbreviation: CES-D, Center for Epidemiologic Studies Depression Scale
[a] No difference at baseline ($P > 0.05$) ; [b] Adjusting for baseline value ; [c] Adjusting for 3-month value

© 2019 Fukuoka Y et al. *JAMA Network Open.*

eTable 3. Safety Data Summary

eTable 3.a. Safety Data Summary: Adverse Events Checklist (All Events) During 3 Month Intervention				
	Total (N=209)	Control (N= 69)	Regular and Plus (N=140)	P value
Person Reporting at least one Event	124 (0.20)	35 (0.17)	89 (0.22)	0.23
**Self Reported Event**	166 (0.27)	49 (0.24)	117 (0.28)	0.30
Heart attack	0 (0.00)	0 (0.00)	0 (0.00)	
Chest pain/angina	1 (0.00)	0 (0.00)	1 (0.00)	0.37
Irregular heartbeats	0 (0.00)	0 (0.00)	0 (0.00)	
Stroke/TIA	0 (0.00)	0 (0.00)	0 (0.00)	
Outpatient surgery	6 (0.01)	3 (0.01)	3 (0.01)	0.39
Fainting/passing out	1 (0.00)	1 (0.00)	0 (0.00)	0.14
Dizziness	13 (0.02)	3 (0.01)	10 (0.02)	0.45
Shortness breath	8 (0.01)	2 (0.01)	6 (0.01)	0.62
Foot pain	43 (0.07)	12 (0.06)	31 (0.07)	0.47
Foot ulcer	0 (0.00)	0 (0.00)	0 (0.00)	
Muscle strain	23 (0.04)	6 (0.03)	17 (0.04)	0.53
Joint stiffness/soreness	47 (0.08)	13 (0.06)	34 (0.08)	0.37
Extreme fatigue	1 (0.00)	0 (0.00)	1 (0.00)	0.37
Back injury	7 (0.01)	3 (0.01)	4 (0.01)	0.58
Fracture	3 (0.00)	1 (0.00)	2 (0.00)	0.99
**Significant medical or mental problems with restricted walking at least 7 consecutive days?**	35	6 (8.6%)	29 (20.0%)	0.05

eTable 3.a. reflects all adverse events (AE) reported by participants during their 1.5 month, 3 month visit, or interim AE events reported prior to 3 month visit. Reported is the number of events and rate per person-month (sum of AE events / sum of follow-up time in month, where follow-up time is AE form date [or actual AE date] minus randomization date). P value is based on Poisson model.

© 2019 Fukuoka Y et al. *JAMA Network Open.*

eTable 3.b. Safety Data Summary: Adverse Events Checklist (All Events) During 6 Month Maintenance					
	Total (N=207)	Control (N= 69)	Regular (N= 70)	Plus (N= 68)	P value
**Person Reporting at least one Event**	159 (0.13)	49 (0.11)	56 (0.14)	54 (0.13)	0.46
**Self Reported Event**	211 (0.17)	60 (0.13)	78 (0.19)	73 (0.18)	0.09
Heart attack	0 (0.00)	0 (0.00)	0 (0.00)	0 (0.00)	
Chest pain/angina	6 (0.00)	2 (0.00)	2 (0.00)	2 (0.00)	1.00
Irregular heartbeats	7 (0.01)	2 (0.00)	2 (0.00)	3 (0.01)	0.84
Stroke/TIA	0 (0.00)	0 (0.00)	0 (0.00)	0 (0.00)	
Outpatient surgery	6 (0.00)	0 (0.00)	2 (0.00)	4 (0.01)	0.06
Fainting/passing out	2 (0.00)	0 (0.00)	2 (0.00)	0 (0.00)	0.12
Dizziness	22 (0.02)	6 (0.01)	11 (0.03)	5 (0.01)	0.30
Shortness breath	9 (0.01)	3 (0.01)	1 (0.00)	5 (0.01)	0.21
Foot pain	41 (0.03)	13 (0.03)	15 (0.04)	13 (0.03)	0.89
Foot ulcer	0 (0.00)	0 (0.00)	0 (0.00)	0 (0.00)	
Muscle strain	37 (0.03)	11 (0.03)	12 (0.03)	14 (0.04)	0.76
Joint stiffness/soreness	46 (0.04)	14 (0.03)	17 (0.04)	15 (0.04)	0.84
Extreme fatigue	20 (0.02)	6 (0.01)	8 (0.02)	6 (0.01)	0.84
Back injury	11 (0.01)	3 (0.01)	4 (0.01)	4 (0.01)	0.90
Fracture	4 (0.00)	0 (0.00)	2 (0.00)	2 (0.00)	0.20
**Significant medical or mental problems with restricted walking at least 7 consecutive days?**	68	21 (26.9%)	23 (30.7%)	24 (32.9%)	0.71

eTable 3.b. reflects all adverse events (AE) reported by participants during their 5 month, 7 month, 9 month visit, or interim AE events reported between 3 month and 9 month visit. Reported is the number of events and rate per person-month (sum of AE events / sum of follow-up time in month, where follow-up time is AE form date [or actual AE date] - month 3 visit date). P value is based on Poisson model.

© 2019 Fukuoka Y et al. *JAMA Network Open.*

eTable 3.c. Safety Data Summary: Adverse Event Checklist (First Event) During 3 Month Intervention				
	Total (N=209)	Control (N= 69)	Regular and Plus (N=140)	P value
Person Reporting at least one Event	101 (0.18)	29 (0.16)	72 (0.20)	0.26
**Self Reported Event**	151 (0.28)	46 (0.25)	105 (0.29)	0.36
Heart attack	0 (0.00)	0 (0.00)	0 (0.00)	
Chest pain/angina	1 (0.00)	0 (0.00)	1 (0.00)	0.37
Irregular heartbeats	0 (0.00)	0 (0.00)	0 (0.00)	
Stroke/TIA	0 (0.00)	0 (0.00)	0 (0.00)	
Outpatient surgery	6 (0.01)	3 (0.01)	3 (0.01)	0.39
Fainting/passing out	1 (0.00)	1 (0.00)	0 (0.00)	0.14
Dizziness	12 (0.02)	3 (0.01)	9 (0.02)	0.56
Shortness breath	8 (0.01)	2 (0.01)	6 (0.01)	0.62
Foot pain	36 (0.06)	10 (0.05)	26 (0.07)	0.49
Foot ulcer	0 (0.00)	0 (0.00)	0 (0.00)	
Muscle strain	21 (0.03)	6 (0.03)	15 (0.04)	0.71
Joint stiffness/soreness	42 (0.07)	12 (0.06)	30 (0.08)	0.44
Extreme fatigue	14 (0.02)	5 (0.02)	9 (0.02)	0.83
Back injury	7 (0.01)	3 (0.01)	4 (0.01)	0.58
Fracture	3 (0.00)	1 (0.00)	2 (0.00)	0.99
**Significant medical or mental problems with restricted walking at least 7 consecutive days?**	29	5 (7.2%)	24 (17.1%)	0.06

eTable 3.c. reflects the first adverse event (AE) reported by participants during their 1.5 month, 3 month visit, or interim AE events reported prior to 3 month visit. Reported is the number of events and rate per person-month (sum of AE events / sum of follow-up time in month, where follow-up time is AE form date [or actual AE date] minus randomization date). P value is based on Poisson model.

© 2019 Fukuoka Y et al. *JAMA Network Open.*

**eTable 3.d. Safety Data Summary: Adverse Event Checklist (First Event) During 6 Month Maintenance**					
	Total (N=207)	Control (N= 69)	Regular (N= 70)	Plus (N= 68)	P value
**Person Reporting at least one Event**	109 (0.11)	31 (0.09)	39 (0.12)	39 (0.12)	0.2957
**Self Reported Event**	190 (0.19)	52 (0.15)	72 (0.23)	66 (0.20)	0.0483
Heart attack	0 (0.00)	0 (0.00)	0 (0.00)	0 (0.00)	
Chest pain/angina	6 (0.00)	2 (0.00)	2 (0.00)	2 (0.00)	0.9992
Irregular heartbeats	7 (0.01)	2 (0.00)	2 (0.00)	3 (0.01)	0.8451
Stroke/TIA	0 (0.00)	0 (0.00)	0 (0.00)	0 (0.00)	
Outpatient surgery	6 (0.00)	0 (0.00)	2 (0.00)	4 (0.01)	0.0577
Fainting/passing out	2 (0.00)	0 (0.00)	2 (0.00)	0 (0.00)	0.1149
Dizziness	21 (0.02)	6 (0.01)	10 (0.02)	5 (0.01)	0.4155
Shortness breath	8 (0.01)	3 (0.01)	1 (0.00)	4 (0.01)	0.3357
Foot pain	38 (0.03)	11 (0.03)	15 (0.04)	12 (0.03)	0.7260
Foot ulcer	0 (0.00)	0 (0.00)	0 (0.00)	0 (0.00)	
Muscle strain	33 (0.03)	9 (0.02)	12 (0.03)	12 (0.03)	0.7101
Joint stiffness/soreness	37 (0.03)	11 (0.03)	14 (0.04)	12 (0.03)	0.8236
Extreme fatigue	19 (0.02)	6 (0.01)	7 (0.02)	6 (0.01)	0.9533
Back injury	10 (0.01)	2 (0.00)	4 (0.01)	4 (0.01)	0.6385
Fracture	3 (0.00)	0 (0.00)	1 (0.00)	2 (0.00)	0.2417
**Significant medical or mental problems with restricted walking at least 7 consecutive days?**	49	12 (17.4%)	18 (25.7%)	19 (27.9%)	0.3065

eTable 3.d. reflects the first adverse event (AE) reported by participants during their 5 month, 7 month, 9 month visit, or interim AE events reported between 3 month and 9 month visit. Reported is the number of events and rate per person-month (sum of AE events / sum of follow-up time in month, where follow-up time is AE form date [or actual AE date] - month 3 visit date). P value is based on Poisson model.

**eTable 3.e. Safety Data Summary: Adverse Event Emergency Department or Urgent Care Facility Visits During 3 Month Intervention**				
	Total (N=209)	Control (N= 69)	Regular and Plus (N=140)	P value
**Total number of emergency department or urgent care facility visits**	12	3	9	
**Total number of participants with emergency department or urgent care facility visits**	11	2	9	
**Total number of emergency department or urgent care facility visits due to an event that occurred during exercise**	1	0	1	
**Relationship between emergency department or urgent care facility visits and intervention**				
Unknown	0	0 (0.0%)	0 (0.0%)	
Not related	11	3 (100%)	8 (88.9%)	0.5465
Possible	1	0 (0.0%)	1 (11.1%)	
Probable	0	0 (0.0%)	0 (0.0%)	
Definite	0	0 (0.0%)	0 (0.0%)	

eTable 3.e. reflects emergency department visits that occurred during the 3 month intervention period, including interim reports. Frequency and percent are shown, where percentages are computed based on the total number of events reported. Only this category had sufficient sample size for statistical analysis. P value is based on Poisson model.

© 2019 Fukuoka Y et al. *JAMA Network Open.*

eTable 3.f. Safety Data Summary: Adverse Events Emergency Department or Urgent Care Facility Visits During 6 Month Maintenance					
	Total (N=207)	Control (N= 69)	Regular (N= 70)	Plus (N= 68)	P value
**Total number of emergency department or urgent care facility visits**	24	5	10	9	
**Total number of participants with emergency department or urgent care facility visits**	23	4	10	9	
**Total number of emergency department or urgent care facility visits due to an event that occurred during exercise**	2	0	2	0	
**Relationship between emergency department or urgent care facility visits and intervention**					
Unknown	0	0 (0.0%)	0 (0.0%)	0 (0.0%)	
Not related	22	5 (100%)	8 (80.0%)	9 (100%)	0.55
Possible	1	0 (0.0%)	1 (10.0%)	0 (0.0%)	
Probable	0	0 (0.0%)	0 (0.0%)	0 (0.0%)	
Definite	0	0 (0.0%)	0 (0.0%)	0 (0.0%)	
eTable 3.f. reflects emergency department visits that occurred between 3 month and 9 month visit, including interim reports during this period. Frequency and percent are shown, where percentages are computed based on the total number of events reported. Only this category had sufficient sample size for statistical analysis. P value is based on Poisson model.					

© 2019 Fukuoka Y et al. *JAMA Network Open.*

eTable 3.g. Safety Data Summary: Adverse Event Hospitalizations During 3 Month Intervention				
	Total (N=209)	Control (N= 69)	Regular and Plus (N=140)	P value
**Total number of hospitalization**	1	0	1	
**Total number of participants hospitalization**	1	0	1	
**Total number of hospitalization due to an event that occurred during exercise**	0	0	0	
**Relationship between hospitalization and intervention**	0	0	0	
Unknown	0	0 (0.0%)	0 (0.0%)	
Not related	1	0 (0.0%)	1 (100%)	
Possible	0	0 (0.0%)	0 (0.0%)	
Probable	0	0 (0.0%)	0 (0.0%)	
Definite	0	0 (0.0%)	0 (0.0%)	
eTable 3.g. reflects hospitalizations that occurred post-randomization during the 3 month intervention period, including interim reports during this period. Frequency and percent are shown, where percentages are computed based on the total number of events reported.				

© 2019 Fukuoka Y et al. *JAMA Network Open.*

eTable 3.h. Safety Data Summary: Adverse Event Hospitalizations During 6 Month Maintenance					
	Total (N=207)	Control (N= 69)	Regular (N= 70)	Plus (N= 68)	P value
**Total number of hospitalization**	5	4	1	0	
**Total number of participants hospitalization**	4	3	1	0	
**Total number of hospitalization due to an event that occurred during exercise**	0	0	0	0	
**Relationship between hospitalization and intervention**	0	0	0	0	
Unknown	0	0 (0.0%)	0 (0.0%)	0 (0.0%)	
Not related	5	4 (100%)	1 (100%)	0 (0.0%)	
Possible	0	0 (0.0%)	0 (0.0%)	0 (0.0%)	
Probable	0	0 (0.0%)	0 (0.0%)	0 (0.0%)	
Definite	0	0 (0.0%)	0 (0.0%)	0 (0.0%)	
eTable 3.h. reflects hospitalizations that occurred post-randomization between 3 month and 9 month visit. Frequency and percent are shown, where percentages are computed based on the total number of events reported.					

eTable 3.i. Safety Data Summary: Adverse Events Checklist (All Events) During 3 Month Intervention				
	Total (N=209)	Control (N= 69)	Regular and Plus (N=140)	P value
Person Reporting at least one Event	124 (0.20)	35 (0.17)	89 (0.22)	0.23
**Self Reported Event**	166 (0.27)	49 (0.24)	117 (0.28)	0.30
Heart attack	0 (0.00)	0 (0.00)	0 (0.00)	
Chest pain/angina	1 (0.00)	0 (0.00)	1 (0.00)	0.37
Irregular heartbeats	0 (0.00)	0 (0.00)	0 (0.00)	
Stroke/TIA	0 (0.00)	0 (0.00)	0 (0.00)	
Outpatient surgery	6 (0.01)	3 (0.01)	3 (0.01)	0.39
Fainting/passing out	1 (0.00)	1 (0.00)	0 (0.00)	0.14
Dizziness	13 (0.02)	3 (0.01)	10 (0.02)	0.45
Shortness breath	8 (0.01)	2 (0.01)	6 (0.01)	0.62
Foot pain	43 (0.07)	12 (0.06)	31 (0.07)	0.47
Foot ulcer	0 (0.00)	0 (0.00)	0 (0.00)	
Muscle strain	23 (0.04)	6 (0.03)	17 (0.04)	0.53
Joint stiffness/soreness	47 (0.08)	13 (0.06)	34 (0.08)	0.37
Extreme fatigue	1 (0.00)	0 (0.00)	1 (0.00)	0.37
Back injury	7 (0.01)	3 (0.01)	4 (0.01)	0.58
Fracture	3 (0.00)	1 (0.00)	2 (0.00)	0.99
**Significant medical or mental problems with restricted walking at least 7 consecutive days?**	35	6 (8.6%)	29 (20.0%)	0.05

eTable 3.i. reflects all adverse events (AE) reported by participants during their 1.5 month, 3 month visit, or interim AE events reported prior to 3 month visit. Reported is the number of events and rate per person-month (sum of AE events / sum of follow-up time in month, where follow-up time is AE form date [or actual AE date] minus randomization date). P value is based on Poisson model.

© 2019 Fukuoka Y et al. *JAMA Network Open.*

eTable 3.j. Safety Data Summary: Adverse Events Checklist (All Events) During 6 Month Maintenance					
	Total (N=207)	Control (N= 69)	Regular (N= 70)	Plus (N= 68)	P value
**Person Reporting at least one Event**	159 (0.13)	49 (0.11)	56 (0.14)	54 (0.13)	0.47
**Self Reported Event**	211 (0.17)	60 (0.13)	78 (0.19)	73 (0.18)	0.09
Heart attack	0 (0.00)	0 (0.00)	0 (0.00)	0 (0.00)	
Chest pain/angina	6 (0.00)	2 (0.00)	2 (0.00)	2 (0.00)	1.00
Irregular heartbeats	7 (0.01)	2 (0.00)	2 (0.00)	3 (0.01)	0.85
Stroke/TIA	0 (0.00)	0 (0.00)	0 (0.00)	0 (0.00)	
Outpatient surgery	6 (0.00)	0 (0.00)	2 (0.00)	4 (0.01)	0.06
Fainting/passing out	2 (0.00)	0 (0.00)	2 (0.00)	0 (0.00)	0.12
Dizziness	22 (0.02)	6 (0.01)	11 (0.03)	5 (0.01)	0.30
Shortness breath	9 (0.01)	3 (0.01)	1 (0.00)	5 (0.01)	0.21
Foot pain	41 (0.03)	13 (0.03)	15 (0.04)	13 (0.03)	0.89
Foot ulcer	0 (0.00)	0 (0.00)	0 (0.00)	0 (0.00)	
Muscle strain	37 (0.03)	11 (0.03)	12 (0.03)	14 (0.04)	0.76
Joint stiffness/soreness	46 (0.04)	14 (0.03)	17 (0.04)	15 (0.04)	0.84
Extreme fatigue	20 (0.02)	6 (0.01)	8 (0.02)	6 (0.01)	0.84
Back injury	11 (0.01)	3 (0.01)	4 (0.01)	4 (0.01)	0.90
Fracture	4 (0.00)	0 (0.00)	2 (0.00)	2 (0.00)	0.20
**Significant medical or mental problems with restricted walking at least 7 consecutive days?**	68	21 (26.9%)	23 (30.7%)	24 (32.9%)	0.71

eTable 3.j. reflects all adverse events (AE) reported by participants during their 5 month, 7 month, 9 month visit, or interim AE events reported between 3 month and 9 month visit. Reported is the number of events and rate per person-month (sum of AE events / sum of follow-up time in month, where follow-up time is AE form date [or actual AE date] - month 3 visit date). P value is based on Poisson model.

© 2019 Fukuoka Y et al. *JAMA Network Open.*

eTable 3.k. Safety Data Summary: Adverse Event Checklist (First Event) During 3 Month Intervention				
	Total (N=209)	Control (N= 69)	Regular and Plus (N=140)	P value
Person Reporting at least one Event	101 (0.18)	29 (0.16)	72 (0.20)	0.26
**Self Reported Event**	151 (0.28)	46 (0.25)	105 (0.29)	0.36
Heart attack	0 (0.00)	0 (0.00)	0 (0.00)	
Chest pain/angina	1 (0.00)	0 (0.00)	1 (0.00)	0.37
Irregular heartbeats	0 (0.00)	0 (0.00)	0 (0.00)	
Stroke/TIA	0 (0.00)	0 (0.00)	0 (0.00)	
Outpatient surgery	6 (0.01)	3 (0.01)	3 (0.01)	0.39
Fainting/passing out	1 (0.00)	1 (0.00)	0 (0.00)	0.14
Dizziness	12 (0.02)	3 (0.01)	9 (0.02)	0.56
Shortness breath	8 (0.01)	2 (0.01)	6 (0.01)	0.62
Foot pain	36 (0.06)	10 (0.05)	26 (0.07)	0.49
Foot ulcer	0 (0.00)	0 (0.00)	0 (0.00)	
Muscle strain	21 (0.03)	6 (0.03)	15 (0.04)	0.71
Joint stiffness/soreness	42 (0.07)	12 (0.06)	30 (0.08)	0.44
Extreme fatigue	14 (0.02)	5 (0.02)	9 (0.02)	0.83
Back injury	7 (0.01)	3 (0.01)	4 (0.01)	0.58
Fracture	3 (0.00)	1 (0.00)	2 (0.00)	0.99
**Significant medical or mental problems with restricted walking at least 7 consecutive days?**	29	5 (7.2%)	24 (17.1%)	0.06
eTable 3.k. reflects the first adverse event (AE) reported by participants during their 1.5 month, 3 month visit, or interim AE events reported prior to 3 month visit. Reported is the number of events and rate per person-month (sum of AE events / sum of follow-up time in month, where follow-up time is AE form date [or actual AE date] minus randomization date). P value is based on Poisson model.				

© 2019 Fukuoka Y et al. *JAMA Network Open.*

eTable 3.l. Safety Data Summary: Adverse Event Checklist (First Event) During 6 Month Maintenance					
	Total (N=207)	Control (N= 69)	Regular (N= 70)	Plus (N= 68)	P value
**Person Reporting at least one Event**	109 (0.11)	31 (0.09)	39 (0.12)	39 (0.12)	0.30
**Self Reported Event**	190 (0.19)	52 (0.15)	72 (0.23)	66 (0.20)	0.05
Heart attack	0 (0.00)	0 (0.00)	0 (0.00)	0 (0.00)	
Chest pain/angina	6 (0.00)	2 (0.00)	2 (0.00)	2 (0.00)	1.00
Irregular heartbeats	7 (0.01)	2 (0.00)	2 (0.00)	3 (0.01)	0.85
Stroke/TIA	0 (0.00)	0 (0.00)	0 (0.00)	0 (0.00)	
Outpatient surgery	6 (0.00)	0 (0.00)	2 (0.00)	4 (0.01)	0.06
Fainting/passing out	2 (0.00)	0 (0.00)	2 (0.00)	0 (0.00)	0.12
Dizziness	21 (0.02)	6 (0.01)	10 (0.02)	5 (0.01)	0.42
Shortness breath	8 (0.01)	3 (0.01)	1 (0.00)	4 (0.01)	0.34
Foot pain	38 (0.03)	11 (0.03)	15 (0.04)	12 (0.03)	0.73
Foot ulcer	0 (0.00)	0 (0.00)	0 (0.00)	0 (0.00)	
Muscle strain	33 (0.03)	9 (0.02)	12 (0.03)	12 (0.03)	0.71
Joint stiffness/soreness	37 (0.03)	11 (0.03)	14 (0.04)	12 (0.03)	0.82
Extreme fatigue	19 (0.02)	6 (0.01)	7 (0.02)	6 (0.01)	0.95
Back injury	10 (0.01)	2 (0.00)	4 (0.01)	4 (0.01)	0.64
Fracture	3 (0.00)	0 (0.00)	1 (0.00)	2 (0.00)	0.24
**Significant medical or mental problems with restricted walking at least 7 consecutive days?**	49	12 (17.4%)	18 (25.7%)	19 (27.9%)	0.31
eTable 3.l. reflects the first adverse event (AE) reported by participants during their 5 month, 7 month, 9 month visit, or interim AE events reported between 3 month and 9 month visit. Reported is the number of events and rate per person-month (sum of AE events / sum of follow-up time in month, where follow-up time is AE form date [or actual AE date] - month 3 visit date). P value is based on Poisson model.					

© 2019 Fukuoka Y et al. *JAMA Network Open.*

**eTable 3.m. Safety Data Summary: Adverse Event Emergency Department or Urgent Care Facility Visits During 3 Month Intervention**				
	Total (N=209)	Control (N= 69)	Regular and Plus (N=140)	P value
**Total number of emergency department or urgent care facility visits**	12	3	9	
**Total number of participants with emergency department or urgent care facility visits**	11	2	9	
**Total number of emergency department or urgent care facility visits due to an event that occurred during exercise**	1	0	1	
**Relationship between emergency department or urgent care facility visits and intervention**				
Unknown	0	0 (0.0%)	0 (0.0%)	
Not related	11	3 (100%)	8 (88.9%)	0.55
Possible	1	0 (0.0%)	1 (11.1%)	
Probable	0	0 (0.0%)	0 (0.0%)	
Definite	0	0 (0.0%)	0 (0.0%)	
eTable 3.m. reflects emergency department visits that occurred during the 3 month intervention period, including interim reports. Frequency and percent are shown, where percentages are computed based on the total number of events reported. Only this category had sufficient sample size for statistical analysis. P value is based on Poisson model.				

© 2019 Fukuoka Y et al. *JAMA Network Open.*

eTable 3.n. Safety Data Summary: Adverse Events Emergency Department or Urgent Care Facility Visits During 6 Month Maintenance					
	Total (N=207)	Control (N= 69)	Regular (N= 70)	Plus (N= 68)	P value
**Total number of emergency department or urgent care facility visits**	24	5	10	9	
**Total number of participants with emergency department or urgent care facility visits**	23	4	10	9	
**Total number of emergency department or urgent care facility visits due to an event that occurred during exercise**	2	0	2	0	
**Relationship between emergency department or urgent care facility visits and intervention**					
Unknown	0	0 (0.0%)	0 (0.0%)	0 (0.0%)	
Not related	22	5 (100%)	8 (80.0%)	9 (100%)	0.55
Possible	1	0 (0.0%)	1 (10.0%)	0 (0.0%)	
Probable	0	0 (0.0%)	0 (0.0%)	0 (0.0%)	
Definite	0	0 (0.0%)	0 (0.0%)	0 (0.0%)	

eTable 3.n. reflects emergency department visits that occurred between 3 month and 9 month visit, including interim reports during this period. Frequency and percent are shown, where percentages are computed based on the total number of events reported. Only this category had sufficient sample size for statistical analysis. P value is based on Poisson model.

© 2019 Fukuoka Y et al. *JAMA Network Open.*

eTable 3.o. Safety Data Summary: Adverse Event Hospitalizations During 3 Month Intervention				
	Total<br>(N=209)	Control<br>(N= 69)	Regular and Plus<br>(N=140)	P value
**Total number of hospitalization**	1	0	1	
**Total number of participants hospitalization**	1	0	1	
**Total number of hospitalization due to an event that occurred during exercise**	0	0	0	
**Relationship between hospitalization and intervention**	0	0	0	
Unknown	0	0 (0.0%)	0 (0.0%)	
Not related	1	0 (0.0%)	1 (100%)	
Possible	0	0 (0.0%)	0 (0.0%)	
Probable	0	0 (0.0%)	0 (0.0%)	
Definite	0	0 (0.0%)	0 (0.0%)	
eTable 3.o. reflects hospitalizations that occurred post-randomization during the 3 month intervention period, including interim reports during this period. Frequency and percent are shown, where percentages are computed based on the total number of events reported.				

© 2019 Fukuoka Y et al. *JAMA Network Open.*

eTable 3.p. Safety Data Summary: Adverse Event Hospitalizations During 6 Month Maintenance					
	Total (N=207)	Control (N= 69)	Regular (N= 70)	Plus (N= 68)	P value
**Total number of hospitalization**	5	4	1	0	
**Total number of participants hospitalization**	4	3	1	0	
**Total number of hospitalization due to an event that occurred during exercise**	0	0	0	0	
**Relationship between hospitalization and intervention**	0	0	0	0	
Unknown	0	0 (0.0%)	0 (0.0%)	0 (0.0%)	
Not related	5	4 (100%)	1 (100%)	0 (0.0%)	
Possible	0	0 (0.0%)	0 (0.0%)	0 (0.0%)	
Probable	0	0 (0.0%)	0 (0.0%)	0 (0.0%)	
Definite	0	0 (0.0%)	0 (0.0%)	0 (0.0%)	
eTable 3.p. reflects hospitalizations that occurred post-randomization between 3 month and 9 month visit. Frequency and percent are shown, where percentages are computed based on the total number of events reported.					

© 2019 Fukuoka Y et al. *JAMA Network Open.*
