# Supplementary material for: Short- and Long-term Effects of a Mobile Phone App in Conjunction With Brief In-Person Counseling on Physical Activity Among Physically Inactive Women: The mPED Randomized Clinical Trial
Source: JAMA Netw Open. 2019 May 24;2(5):e194281. doi: 10.1001/jamanetworkopen.2019.4281 (PMC6632135; doi:10.1001/jamanetworkopen.2019.4281)
Supplement: Supplement 3. — Data Sharing Statement [file jamanetwopen-2-e194281-s003.pdf]

Data Sharing Statement

Fukuoka Y, Haskell W, Lin F, Vittinghoff E. Short- and long-term effects of a mobile phone app in conjunction with brief in-person counseling on physical activity among physically inactive women: the mPED randomized clinical trial. *JAMA Netw Open*. 2019;2(5):e194281. doi:10.1001/jamanetworkopen.2019.4281

Data

Data available: *No*
